# Effects of the therapeutic correction of U1 snRNP complex on Alzheimer’s disease

**DOI:** 10.1038/s41598-024-81687-2

**Published:** 2024-12-03

**Authors:** Caio Bruno Q. S. Leal, Camila G. M. Zimmer, Vanessa V. C. Sinatti, Ericks S. Soares, Britt Poppe, Adrien Carton de Wiart, Xue Ying Chua, Ronan V. da Silva, Margaret H. Magdesian, Michael S. Rafii, Luc Buée, Rafael M. Bottos

**Affiliations:** 1 Aptah Bio Inc., MBC BioLabs, 930 Brittan Avenue, San Carlos, 94070 USA; 2 Ananda Devices Inc., Laval, H7V 5B7 Canada; 3 Alzheimer’s Therapeutic Research Institute, University of Southern California, San Diego, 92121 USA; 4 Alzheimer and Tauopathies, CHU-Lille, INSERM, University of Lille, Lille, 59000 France; 5 Vesper Biotechnologies, Dover, LP 19904 USA

**Keywords:** Alzheimer’s disease, Astrogliosis, TAU, Amyloid-beta, U1-70K, U1 snRNP, RNA, Biotechnology, Drug discovery, Alzheimer's disease

## Abstract

The U1 snRNP complex recognizes pre-mRNA splicing sites in the early stages of spliceosome assembly and suppresses premature cleavage and polyadenylation. Its dysfunction may precede Alzheimer’s disease (AD) hallmarks. Here we evaluated the effects of a synthetic single-stranded cDNA (APT20TTMG) that interacts with U1 snRNP, in iPSC-derived neurons from a donor diagnosed with AD and in the SAMP8 mouse model. APT20TTMG effectively binds to U1 snRNP, specifically decreasing TAU in AD neurons, without changing mitochondrial activity or glutamate. Treatment enhanced neuronal electrical activity, promoted an enrichment of differentially expressed genes related to key processes affected by AD. In SAMP8 mice, APT20TTMG reduced insoluble pTAU in the hippocampus, amyloid-beta and GFAP in the cortex, and U1-70 K in both brain regions, without cognitive changes. This study highlights the correction of the U1 snRNP complex as a new target for AD.

**Supplementary Information:**

The online version contains supplementary material available at 10.1038/s41598-024-81687-2.

## Introduction

The U1 small nuclear ribonucleoprotein (snRNP) complex is one of the main complexes involved in splicing^1^. It recognizes pre-mRNA splicing sites during the early stages of spliceosome assembly, attracting other snRNPs and completing spliceosome assembly and splicing^1^. U1 snRNP also actively suppresses premature 3′-end cleavage and polyadenylation through the telescripting process, using early polyadenylation signals that would lead to aberrant and truncated mRNAs^2,3^.

In Alzheimer’s disease (AD), U1 snRNP dysfunction causes cytoplasmic aggregation and mRNA splicing deficiency^4^, leading to incorrect splicing and enhanced premature cleavage and polyadenylation, and could be related to neuronal cell cycle re-entry due to cytoplasmic translocation^5–7^. Spliceosome components are associated with TAU protein and cryptic splicing load is increased in human brains with TAU pathology^8^. This suggests that TAU aggregation is correlated with spliceosome cytoplasmic sequestration of spliceosome components and disruption of snRNPs assembly and stability. Moreover, U1-70 K interacts with TAU and both U1-70 K and TAU co-localize to neurofibrillary tangles (NFTs)^9,10^. Notably, U1-70 K is detected before NFTs^9^, indicating that U1 snRNP aggregation likely precedes TAU aggregation, enhancing spliceosome dysfunction.

U1 snRNP components, predominantly U1-70 K and U1-A proteins, aggregate and form tangle-like structures in AD^7^. Also, both proteins are strongly correlated with insoluble amyloid-beta (Aβ) and TAU^11^. However, current therapies primarily target the removal of toxic proteins, disregarding this common underlying mechanistic origin.

Since U1 snRNP represents a promising therapeutic target in AD, here we evaluated the effects of a synthetic single-stranded cDNA, named APT20TTMG (patent no. US11946050B2), in human induced pluripotent stem cells (iPSC)-derived neurons from a donor diagnosed with AD and in the Senescence Accelerated Mouse-Prone (SAMP8) model. APT20TTMG has strategic sequence, structure, and chemical modifications to bind to U1 snRNP and pre-mRNAs conserved regions, ensuring the correct assembly during the splicing initiation process of all transcripts, without silencing or inhibiting genes. This study highlights the critical role of the U1 snRNP complex in AD, demonstrating that the correction of U1 snRNP function induces beneficial changes in the expression profile of important genes, while specifically reducing TAU levels in AD neurons. Furthermore, it leads to reduced levels of Aβ and insoluble pTAU proteins across multiple brain areas, and mitigates severe astrogliosis in the SAMP8 mice.

## Results

### APT20TTMG interacts with U1 snRNP components

**Fig. 1:**
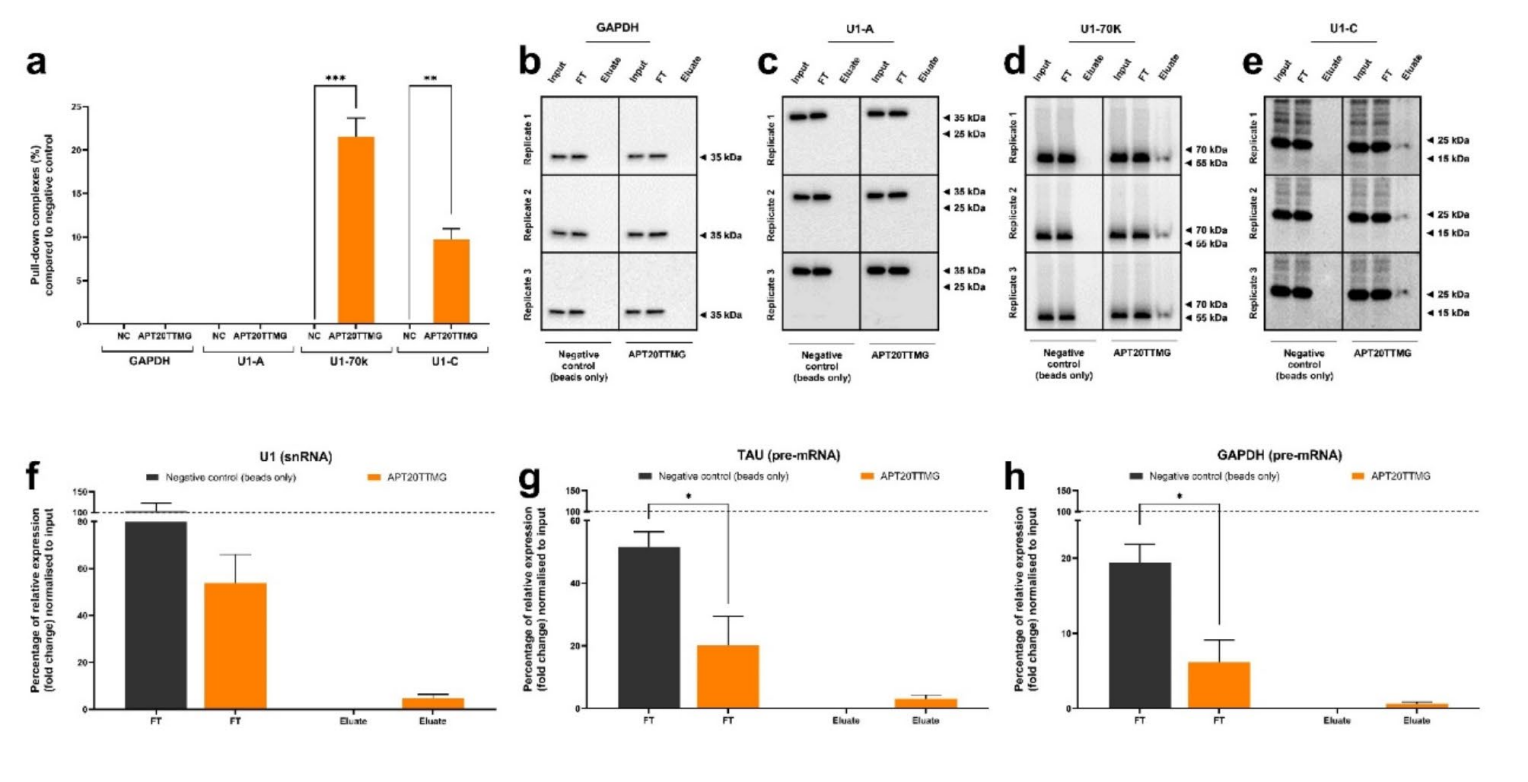
APT20TTMG selectively binds to U1 snRNP complex, U1 snRNA, and pre-mRNAS. Human neuroblastoma SK-N-SH cells were lysed and prepared for protein immunoprecipitation, with quantifications normalized in relation to input (100%), and pull-down assays, performed with RNA isolated from input (50%), flow-through - FT (50%), and eluate (100%) samples. Percentage of assembly to GAPDH, U1-A, U1-70 K, and U1-C (**a**). Western blot representative images of GAPDH, U1-A, U1-70 K, and U1-C (**b**–**e**). Relative expression of U1 snRNA, TAU, and GAPDH pre-mRNAs (**f**–**h**). Data are represented as mean ± SEM. Graphs show data from three independent experiments, analyzed using ANOVA, followed by Tukey’s post hoc test, or t-test. *P < 0.05, **P < 0.01, and ***P < 0.001.

To confirm the binding of APT20TTMG to snRNPs in the U1 complex and pre-mRNAs during complex assembly, pull down assays were carried out using SK-N-SH neuroblastoma cells. As shown in Fig. 1a–e, APT20TTMG specifically binds to U1-70 K and U1-C, but not to U1-A snRNP. Although no statistical difference is observed in relation to the negative control, the elution fraction from the pull-down assay in the presence of APT20TTMG indicates an interaction with the U1 snRNA and TAU and GAPDH pre-mRNAs (Fig. 1f–h and Supplementary Fig. 1). Additionally, there is a statistical reduction in the flow-through compared to the negative control for TAU and GAPDH pre-mRNAs. Original blots are presented in Supplementary Fig. 2.

### APT20TTMG enhances electrical activity of neurons, tends to improve neuronal morphology, and specifically decreases TAU in AD neurons without affecting mitochondrial activity or glutamate levels

**Fig. 2:**
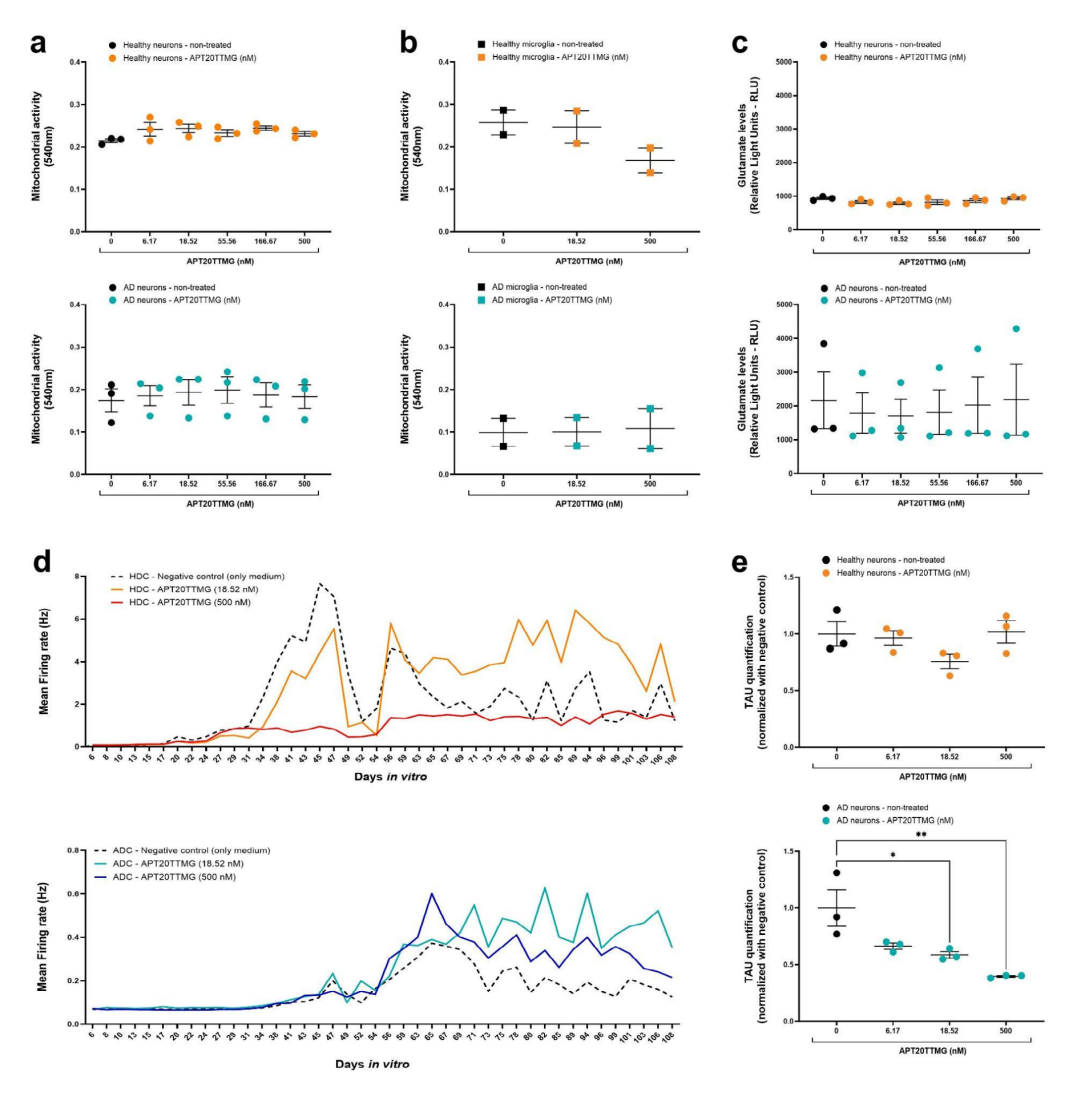
APT20TTMG does not affect mitochondrial activity in iPSC-derived neurons and microglia, or glutamate in neurons, while enhances spontaneous neuronal electrical activity and specifically decreases TAU levels in iPSC-derived AD neurons. iPSC-derived healthy and AD neurons and microglia, cultured for 6 days, were treated with different concentrations of APT20TTMG (neurons: 6.17, 18.52, 55.56, 166.67, or 500 nM; microglia:18.52 or 500 nM), for 7 days. Mitochondrial activity of neurons (**a**) and microglia (**b**), measured by the MTT assay. Glutamate levels in iPSC-derived neurons (**c**), measured by the Glutamate-Glo™ Assay. For the analysis of neuronal electrical activity, iPSC-derived neurons cultured for 6 days were treated with two concentrations of APT20TTMG (18.52 nM or 500 nM), three times per week, and the electrophysiological measurements were performed 4 h before treatments, with a total recording time of 5 min. The spontaneous firing was performed 2 min before and after stimulation, and the spontaneous activity was recorded from days six to 108. Mean firing rate of healthy neurons (upper: dotted, orange, and red lines represent medium (negative control), 18.52 nM APT20TTMG, and 500 nM APT20TTMG, respectively) and AD neurons (lower: dotted, light blue, and dark blue lines represent medium (negative control), 18.52 nM APT20TTMG, and 500 nM APT20TTMG, respectively) (**d**). TAU protein levels were evaluated by the Human Tau SimpleStep ELISA (**e**). Data are represented as mean ± SEM or mean firing rate (Hz). Graphs show data from three independent experiments performed in three technical replicates, for mitochondrial activity and TAU levels in neurons, two independent experiments performed in three technical replicates for mitochondrial activity in microglia, three independent experiments performed in six technical replicates for glutamate release, and one biological replicate performed in six technical replicates for electrophysiology, analyzed using ANOVA, followed by Tukey’s post hoc test (graphs a-d) or Dunnett’s post hoc test (graph e). *P < 0.05 and **P < 0.01.

To verify the impact of APT20TTMG on cellular processes such as mitochondrial function and glutamate regulation, we conducted assays on iPSC-derived neurons and microglia. The mitochondrial activity was not changed in neurons and microglia (Fig. 2a, b, respectively), suggesting that APT20TTMG is not toxic to both neuronal and non-neuronal cells. Higher levels of glutamate observed in AD neurons, compared to healthy neurons (Fig. 2c), could suggest a glutamate-mediated excitotoxicity. Although no significant difference was observed, lower concentrations of APT20TTMG tended to decrease glutamate release in AD neurons and did not promote changes in healthy neurons.

Given that AD progression is closely correlated with morphological neuronal changes, the fiber breadth, axonal material, axonal branching, and branch junctions parameters were investigated in over 1,000 neurons per condition in NeuroHTS™ microplates, enabling a comprehensive assessment of healthy and AD neurons treated with 18.52 nM APT20TTMG in relation non-treated neurons. In addition to a trend towards an increase in the fiber breadth of healthy neurons, treated AD neurons showed a similar fiber breadth profile to non-treated healthy neurons (Supplementary Fig. 3b, c). Although there were no statistical differences, APT20TTMG (18.52 and 56.66 nM) increased approximately 80% the number of axonal material in healthy neurons. Similarly, AD neurons treated with 18.52 nM APT20TTMG presented a statistically non-significant increase of approximately 50% in axonal branching (Supplementary Fig. 3d). Despite a high variability, 6.17 nM and 18.52 nM APT20TTMG tended to increase the number of branch junctions by approximately 2 and 3.5 times, respectively (Supplementary Fig. 3e). Also, AD neurons presented 65% fewer branch junctions than non-treated healthy neurons (Supplementary Fig. 3f).

To analyze the electrical activity, the concentrations of 18.52 and 500 nM APT20TTMG were tested. In healthy neurons, firing was detected after a few days in culture, while in AD neurons it started to be detected only after 40 days (Fig. 2d), being lower than that of healthy neurons. Healthy and AD neurons treated with 18.52 nM APT20TTMG had an increase of approximately 29.9% and 69.1%, respectively, in their electrical activity. However, 500 nM appears to impair the electrical activity in healthy neurons, while improving it in AD neurons.

As TAU is highly affected in AD neurons, we investigated whether treatment with APT20TTMG could change its levels in iPSC-derived neurons by ELISA. Treatment with 6.17, 18.52, and 500 nM APT20TTMG did not change TAU levels in healthy neurons (Fig. 4b). However, these same concentrations reduced TAU by 33.7 ± 2.7%, 41.4 ± 2.9% (*P* = 0.0196), and 60.4 ± 0.8% (*P* = 0.0023), respectively, in AD neurons.

### APT20TTMG induces beneficial changes in gene expression profile

For an overall analysis of differential gene expression and key pathways involved in neuronal changes observed after treatment of AD neurons with APT20TTMG, we performed a RNA sequencing (RNA-Seq) data analysis. The comparison between non-treated healthy and AD neurons shows the highest numbers of differentially expressed genes (DEGs), representing 10,251, 1107 with log2FoldChange of two, and 179 with log2FoldChange of four (Table 1 and Supplementary Table 1), as shown in the heatmap and volcano plots (Supplementary Figs. 4 and 5, respectively). AD neurons treated with 18.52 nM APT20TTMG presented 602 DEGs when compared to non-treated AD neurons, with an absolute log2FoldChange value of less than two (Table 1, Supplementary Tables 2, and Supplementary Figs. 4 and 5). The overlap of DEGs between both comparisons is illustrated in the Venn diagram (Supplementary Fig. 6), highlighting 76 shared downregulated genes and 68 shared upregulated genes. Enrichment analysis of DEGs related to biological processes (GO biological processes – GO: BP) was associated with critical signaling pathways such as the generation of neurons, neuron differentiation, cell morphogenesis, and regulation of synapsis assembly in AD neurons treated with 18.52 nM APT20TTMG, compared to non-treated AD neurons. This enrichment profile was similar to the non-treated healthy and AD neurons comparison (Supplementary Tables 3 and 4).

**Table 1:** Number of differentially expressed genes in each pairwise comparison, considering the adjusted p-value (cutoff of 0.05) and log2 fold change.

Treated AD neurons (18.52 nM) vs. Non-treated AD neurons			Non-treated healthy neurons vs. Non-treated AD neurons		
LFC	padj < 0.05	padj < 0.01	LFC	padj < 0.05	padj < 0.01
*All*	602	332	*All*	10,251	8424
*FC > 2*	0	0	*FC > 2*	1107	1107
*FC > 4*	0	0	*FC > 4*	179	179
*FC > 8*	0	0	*FC > 8*	53	53

AD: Alzheimer’s disease; FC: Fold change; LFC: Log fold change; padj: Adjusted p value.

**Fig. 3:**
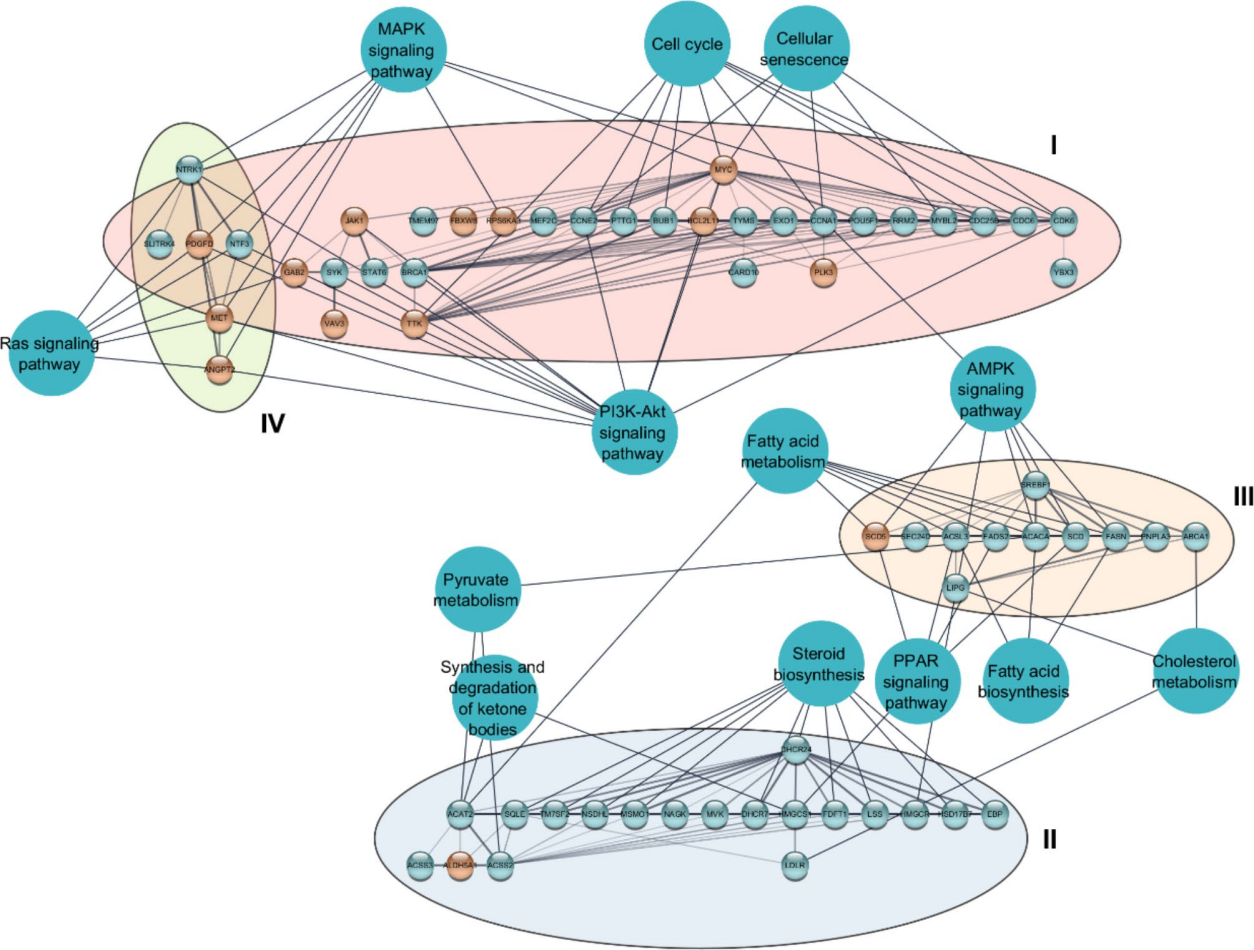
APT20TTMG induces beneficial changes in gene expression profile in iPSC-derived neurons. Protein-protein interaction network and clustering obtained from enrichment analysis of metabolic pathways of differentially expressed genes (DEGs) in AD neurons treated with 18.52 nM APT20TTMG using STRING and AutoAnnotate apps from Cytoscape. Inside each cluster, the nodes represent protein products of the investigated DEGs and the edges denote protein-protein interactions. The blue nodes represent the main enriched metabolic pathways related to the protein clusters. Blue and red circles represent upregulated and downregulated genes, respectively. Cluster I: cell cycle (FDR value 2.95E-7, 12 DEGs), PI3K-Akt signaling (FDR value 3.43E-6, 17 DEGs), and cellular senescence (FDR value 5.65E-5, 10 DEGs) pathways. Cluster II: steroid biosynthesis (FDR value 3.64E-11, 10 DEGs), synthesis and degradation of ketone bodies (FDR value 0.0171, two DEGs), and pyruvate metabolism (FDR value 0.018, three DEGs) pathways. Cluster III: fatty acid metabolism (FDR value 3.7E-5, seven DEGs) and biosynthesis (FDR value 0.004, three DEGs), cholesterol metabolism (FDR value 0.0063, four DEGs), AMPK (FDR value 3.71E-9, 14 DEGs), and PPAR (FDR value 9.2E-4, six DEGs) signaling pathways. Cluster IV: MAPK (FDR value 7.45E-9, 19 DEGs) and Ras (FDR value 5.54E-5, 12 DEGs) signaling pathways.

The metabolic pathway enrichment and network analysis identified four prominent clusters containing more than six DEGs (Fig. 3). They were predominantly associated with cell cycle (FDR value 2.95E-7, 12 DEGs), phosphoinositide 3-kinase/serine-threonine kinase (PI3K-Akt) (FDR value 3.43E-6, 17 DEGs), and cellular senescence (FDR value 5.65E-5, 10 DEGs) (cluster I; Supplementary Table 5); steroid biosynthesis (FDR value 3.64E-11, 10 DEGs), synthesis and degradation of ketone bodies (FDR value 0.0171, two DEGs), and pyruvate metabolism (FDR value 0.018, three DEGs) (cluster II; Supplementary Table 5); fatty acid metabolism (FDR value 3.7E-5, seven DEGs) and biosynthesis (FDR value 0.004, three DEGs), cholesterol metabolism (FDR value 0.0063, four DEGs), AMP-activated protein kinase (AMPK) (FDR value 3.71E-9, 14 DEGs), and peroxisome proliferator-activated receptor (PPAR) (FDR value 9.2E-4, six DEGs) signaling pathways (cluster III; Supplementary Table 5); and mitogen-activated protein kinase (MAPK) (FDR value 7.45E-9, 19 DEGs) and Ras (FDR value 5.54E-5, 12 DEGs) signaling pathways (cluster IV; Supplementary Table 5).

Main DEGs observed in these neurons, their associations with the disease, and suggested roles in the mechanism of action of APT20TTMG, are detailed in Supplementary Fig. 7. This involves U1 snRNP recruitment and splicing regulation, impacting pre-mRNA processing, and leading to homeostasis of key events such as cell cycle regulation, mRNA translation, and pathways of transport and catabolism. These events result in reduced neurodegeneration, offering neuroprotection.

### A low dose of APT20TTMG did not affect the cognition of SAMP8 mice

To investigate whether 42 days of treatment with APT20TTMG affects cognitive function, SAMP8 mice were evaluated in the Morris water maze (MWM) and contextual fear conditioning (CFC) behavioral tests. During the four-day training session of the MWM test, animals treated with 0.3 µg/day APT20TTMG exhibited a performance similar to the vehicle group, with reduced escape latency, total swimming distance, and thigmotaxis, accompanied by a slight increase in floating behavior over time (Supplementary Fig. 8a-e). However, animals treated with the highest dose (3 µg/day) showed an increase of 83.51 ± 12.40% (*P* < 0.0001) and 56.73 ± 12.97% (*P* = 0.0046) in the escape latency on days 3 and 4, respectively, in addition to an increase in the swimming distance, also on days 3 (66.41 ± 10.58%, *P* = 0.0008) and 4 (46.37 ± 13.31%, *P* = 0.036). This highest dose increased floating behavior by 51.35 ± 12.76% only on day 3 (*P* = 0.0168), when compared to the vehicle group. During the probe trial, animals treated with both doses of APT20TTMG showed a number of target zone crossings and a percentage of time spent in the target quadrant similar to the vehicle group (Supplementary Fig. 8f, g), although the highest dose has shown a downward trend.

In the sixth week of treatment, animals were evaluated in the CFC test. No differences were observed in the freezing behavior over time among all groups (Supplementary Fig. 8h).

Additionally, all animals exhibited a slight body weight loss during the first week after the pump implantation (Supplementary Fig. 8i). Although body weights largely stabilized in the subsequent weeks, mice treated with 3 µg/day APT20TTMG showed slightly but significantly reduced body weights compared to vehicle-treated animals. Considering these data, we proceeded with further experiments using the low dose of 0.3 µg/day.

### APT20TTMG corrects the U1 snRNP complex of SAMP8 mice brains

**Fig. 4:**
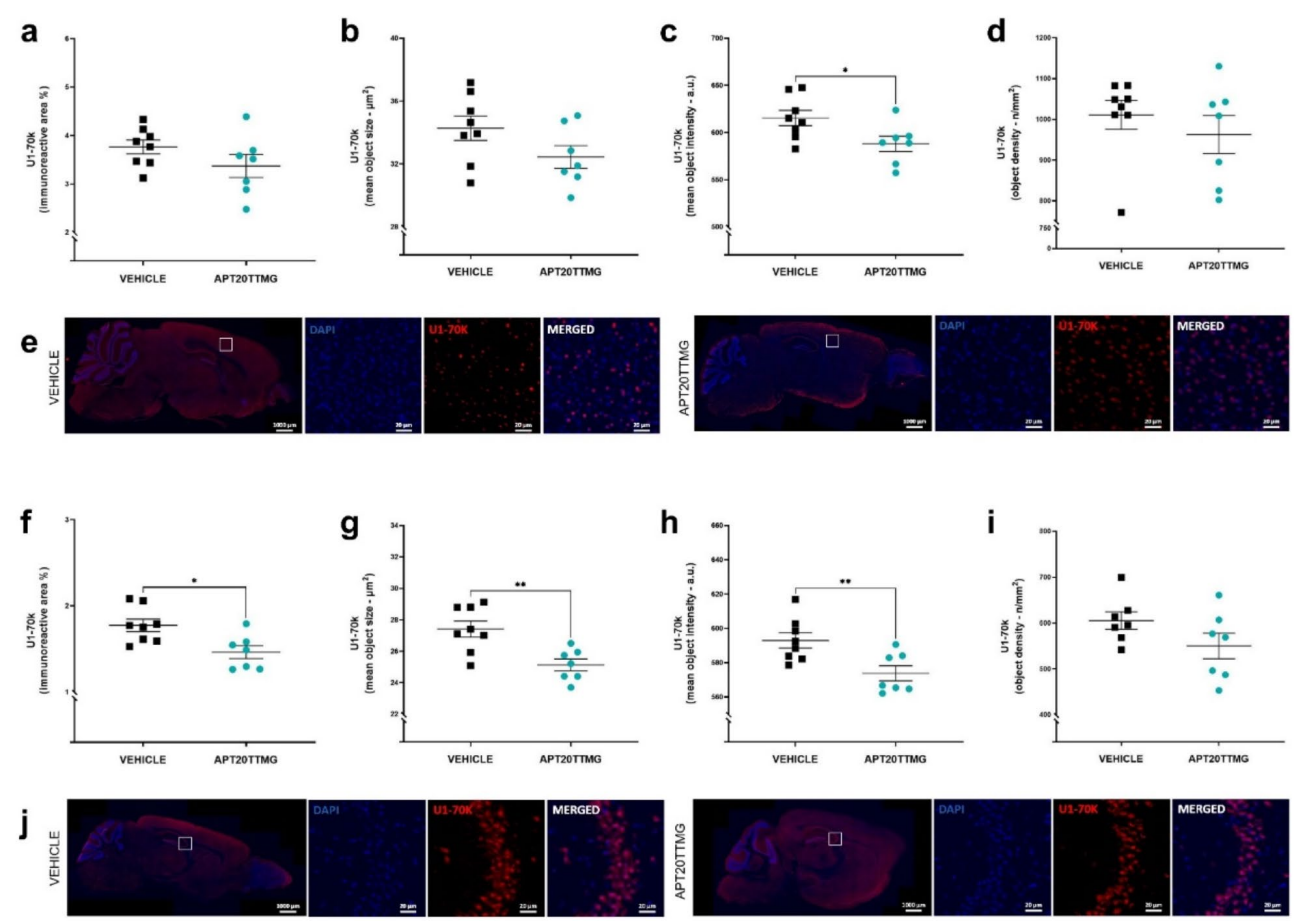
APT20TTMG decreases U1-70 K immunofluorescence in the cerebral cortex and hippocampus of SAMP8 mice. Following a 42-day treatment with 0.3 µg/day APT20TTMG or vehicle, female SAMP8 mice brains were collected and the cortex and hippocampus were processed for U1-70 K immunohistochemistry. Immunoreactive area (**a**), mean object size (**b**), mean object intensity (**c**), mean object density (**d**) in cortex, and representative image of cortex, double-labeled with U1-70 K (cytoplasm) and DAPI (nucleus) (**e**). Immunoreactive area (**f**), mean object size (**g**), mean object intensity (**h**), mean object density (**i**) in hippocampus, and representative image of hippocampus, double-labeled with U1-70 K (cytoplasm) and DAPI (nucleus) (**j**). The means of immunofluorescent signal were measured within the regions of interest (ROI) on 5 brain sections per mouse and data are represented as mean ± SEM (*n* = 7–8). Graphs were analyzed using T-test (a, b, c, f, g, h, and i) or Mann-Whitney test (d), comparing all groups to vehicle-treated control animals. *P < 0.05 and **P < 0.01.

Since APT20TTMG was designed to target the U1 snRNP complex and correct its function, we investigated possible alterations in one of its main components, U1-70 K, in the cortex and hippocampus of SAMP8 mice. As shown in Fig. 4, animals treated with APT20TTMG had reduced levels of this protein in both tissues.

In the cortex of animals treated with 0.3 µg/day APT20TTMG (Fig. 4a–e), U1-70 K mean object intensity (Fig. 4c) was reduced by 4.44 ± 1.33% when compared to vehicle-treated animals (*P* = 0.034). In their hippocampus (Fig. 4f-j), the immunoreactive area, mean object size, and mean object intensity (Fig. 4f-h) were also significantly reduced by 17.57 ± 4.24% (*P* = 0.0108), 8.32 ± 1.38% (*P* = 0.0039), and 3.23 ± 0.74% (*P* = 0.0096), respectively. It may indicate that APT20TTMG could reduce the aggregation and cytoplasmic mislocalization of U1-70 K, commonly observed in AD.

### APT20TTMG reduces levels of TAU and Aβ in the brains of SAMP8 mice

SAMP8 mice brains were fractionated to determine whether APT20TTMG treatment would affect soluble and insoluble TAU and pTAU proteins levels. Additionally, we verified differences in the immunofluorescence for pTAU and Aβ in target regions of the brains.

**Fig. 5:**
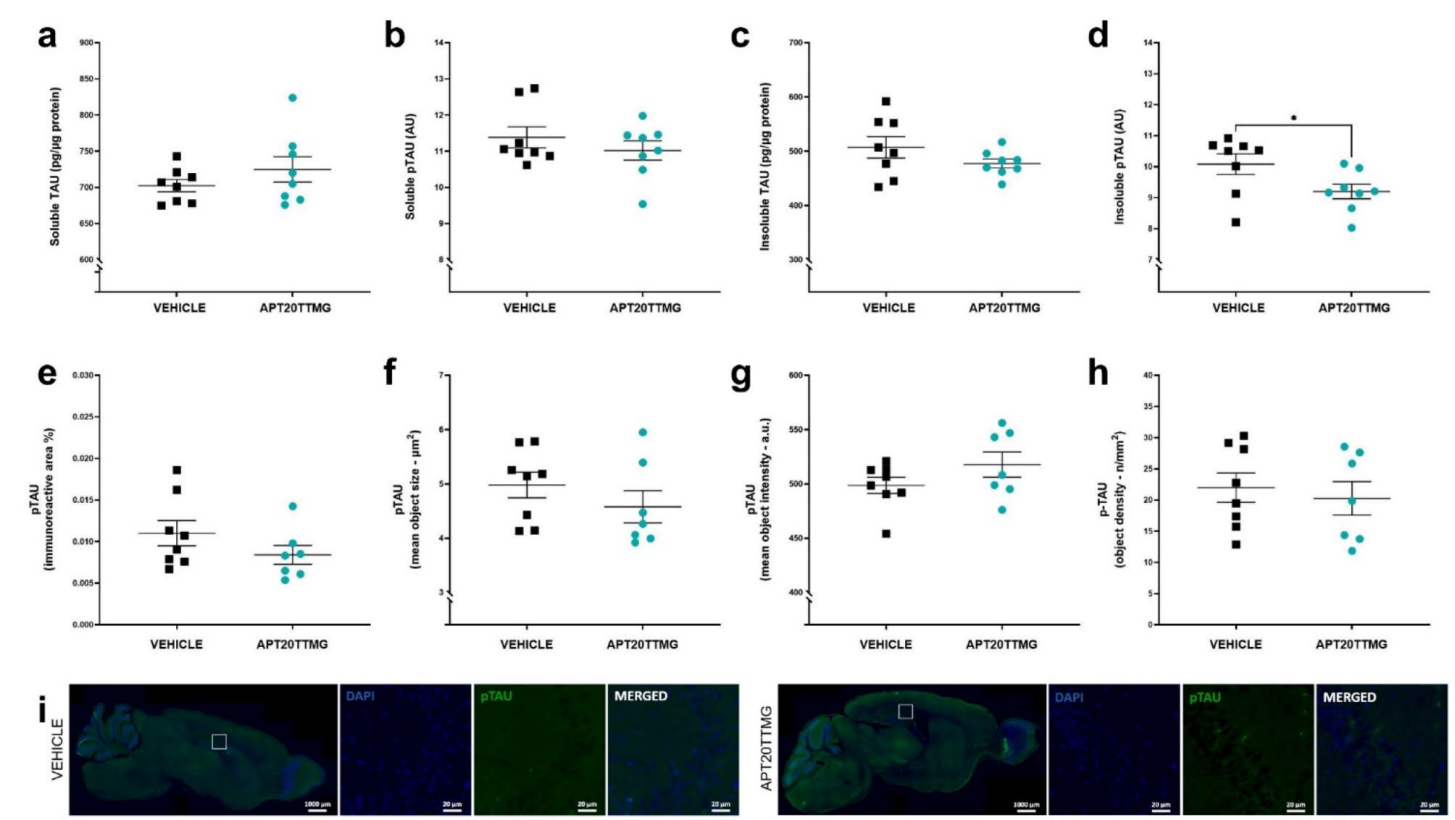
APT20TTMG specifically decreases levels of insoluble pTAU in the hippocampus of SAMP8 mice. Following a 42-day treatment with 0.3 µg/day APT20TTMG or vehicle, female SAMP8 mice brains were collected and the hippocampus processed for ELISA and immunohistochemistry analysis. Levels of soluble TAU (**a**), soluble pTAU (T231) (**b**), insoluble TAU (**c**), and insoluble pTAU (T231) (**d**) in the hippocampus were measured by ELISA. Immunoreactive area (**e**), mean object size (**f**), mean object intensity (**g**), mean object density (**h**) for pSer202/pThr205-TAU in the hippocampus, and representative image of hippocampus, double-labeled with pTAU (cytoplasm) and DAPI (nucleus) (**i**). For ELISA, data are represented as mean ± SEM (*n* = 8). The means of immunofluorescent signal were measured within the regions of interest (ROI) on 5 brain sections per mouse and data are represented as mean ± SEM (*n* = 7–8). Graphs were analyzed using t-test (a, c, d-h) or Mann-Whitney test (b), comparing all groups to vehicle-treated control animals. *P < 0.05.

Animals did not present any changes in total TAU and pTAU231 levels in soluble and insoluble fractions of the cortex (Supplementary Fig. 9a-d). Nevertheless, a significant reduction of 8.76 ± 2.32% (*P* = 0.0476) in insoluble pTAU231 levels was observed in the hippocampus upon treatment with 0.3 µg/day APT20TTMG (Fig. 5d), while soluble/insoluble TAU and soluble pTAU231 remained unchanged in the same region (Fig. 5a-c). Immunofluorescence for pSer202/pThr205-TAU content was not altered by the treatment in the cortex (Supplementary Fig. 9e-i) and hippocampus (Fig. 5e-i). Despite the lack of statistical significance, there was a tendency for a decrease in the immunoreactive area in the hippocampus (23.64 ± 10.33%) after treatment with APT20TTMG (Fig. 5e), similar to the significant reduction pattern of insoluble pTAU levels in the same area.

**Fig. 6:**
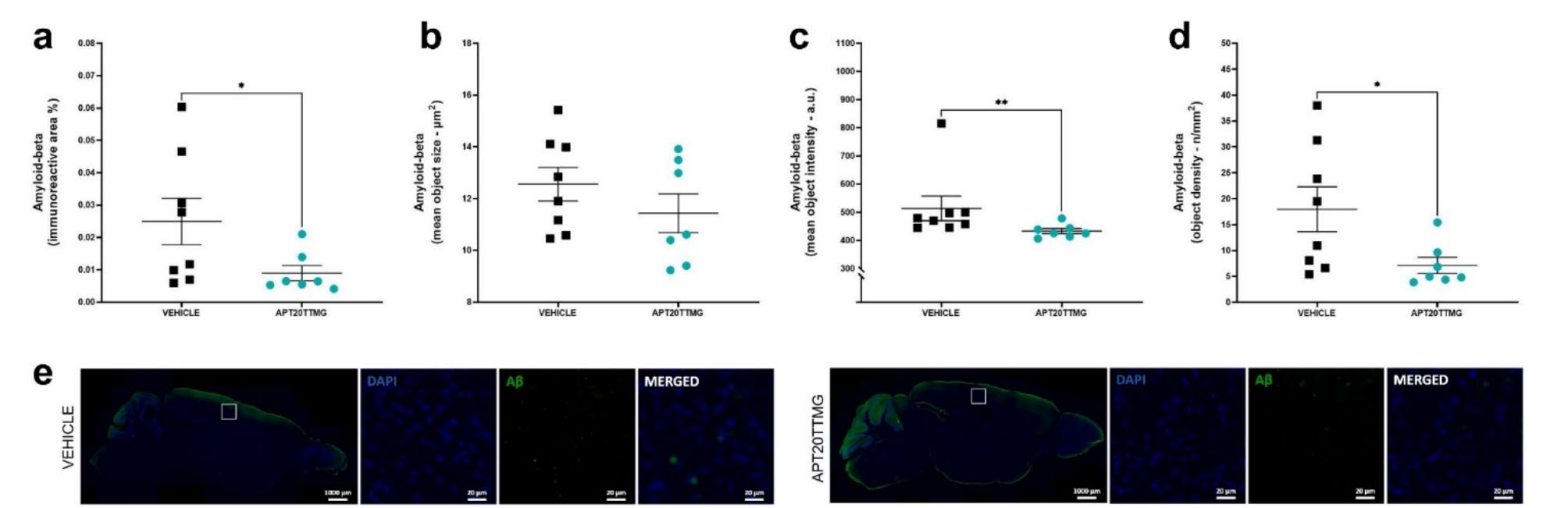
APT20TTMG decreases Aβ immunofluorescence in the cerebral cortex of SAMP8 mice. Following a 42-day treatment with 0.3 µg/day APT20TTMG or vehicle, female SAMP8 mice brains were collected and the cortex processed for immunohistochemistry analysis. Immunoreactive area (**a**), mean object size (**b**), mean object intensity (**c**), mean object density (**d**) in the cerebral cortex, and representative image of cortex, double-labeled with Aβ (cytoplasm) and DAPI (nucleus) (**e**). The means of immunofluorescent signal were measured within the regions of interest (ROI) on 5 brain sections per mouse and data are represented as mean ± SEM (*n* = 7–8). Graphs were analyzed using Mann-Whitney test (a and c) or T-test (b and d), comparing all groups to vehicle-treated control animals. *P < 0.05 and **P < 0.01.

When compared with the vehicle group, animals treated with APT20TTMG showed reduced immunoreactive area for Aβ by 64.04 ± 9.39% (*P* = 0.0401) (Fig. 6a). A similar reduction was observed in object density (60.18 ± 8.73%, *P* = 0.0448) (Fig. 6d) and a lower reduction in the mean object intensity (15.66 ± 1.74%, *P* = 0.0037) (Fig. 6c). Treatment did not change the mean object size of the protein in the cortex (Fig. 6b) nor any evaluated parameters in the hippocampus of the animals (Supplementary Fig. 10).

These results indicate that APT20TTMG specifically decreases levels of insoluble pTAU in the hippocampus and Aβ in the cortex. This effect may be associated with reduced levels of U1-70 K in both brain regions, which supports the hypothesis that U1 snRNP dysfunction is an upstream event that leads to the development of key hallmarks of AD pathology.

### APT20TTMG controls severe astrogliosis in SAMP8 mice

**Fig. 7:**
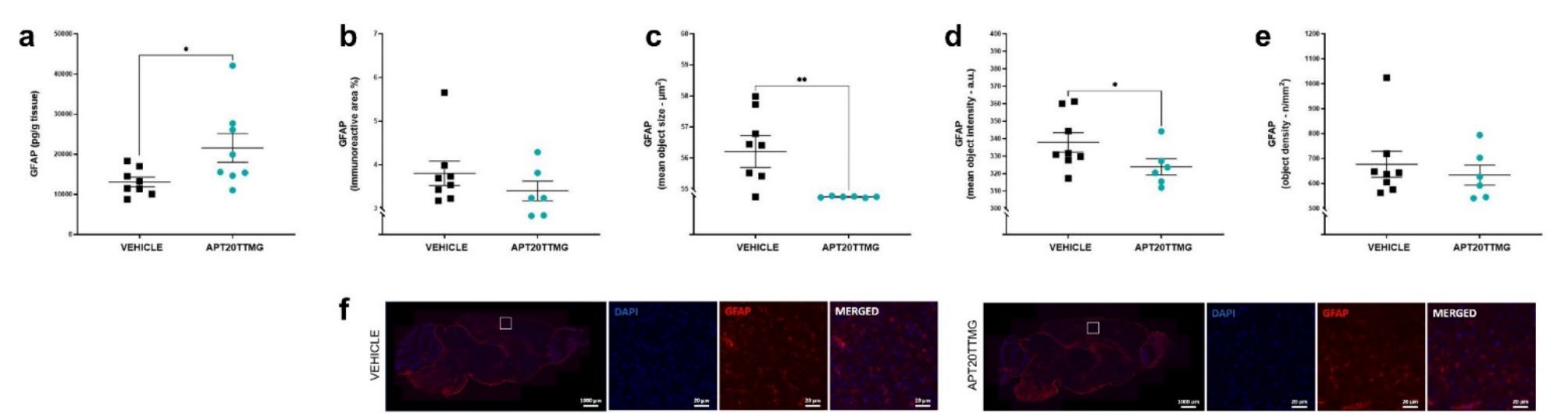
APT20TTMG increases soluble GFAP levels and decreases GFAP immunofluorescence in the cerebral cortex of SAMP8 mice. Following a 42-day treatment with 0.3 µg/day APT20TTMG or vehicle, female SAMP8 mice brains were collected. Levels of GFAP were measured by ELISA, in the soluble fraction of the cortex, and by immunohistochemistry. GFAP levels in the soluble fraction (**a**), immunoreactive area (**b**), mean object size (**c**), mean object intensity (**d**), mean object density (**e**) of GFAP immunofluorescence in the cortex, and representative image of cortex, double-labeled with GFAP (cytoplasm) and DAPI (nucleus) (**f**). Samples for ELISA were collected from 8 animals per treatment, and the means of immunofluorescent signal were measured within the regions of interest (ROI) on 5 brain sections per mouse. Data are represented as mean ± SEM (*n* = 6–8). Graphs were analyzed using T-test (a-c, e) or Mann-Whitney test (d), comparing all groups to vehicle-treated control animals. *P < 0.05 and **P < 0.01.

The functional, molecular, and morphological remodeling of astrocytes, known as reactive astrogliosis, is another key characteristic of AD. This phenomenon leads to an increase in the astrocyte marker glial fibrillary acidic protein (GFAP). To quantify its levels, we analyzed the soluble fraction and cryosections of the cortex and hippocampus. GFAP levels were significantly elevated in the soluble fraction of the cortex of APT20TTMG-treated animals compared to vehicle-treated animals by 64.50 ± 27.24% (*P* = 0.0412) (Fig. 7a). On another hand, treatment triggered a reduction of 4.99 ± 0.68% in the mean object size (*P* = 0.0015) and 4.16 ± 1.37% in the mean object intensity (*P* = 0.0426) for GFAP immunofluorescence (Fig. 7c, d, respectively). APT20TTMG appears to have a tissue-specific effect, since it did not alter GFAP in any analysis in the hippocampus (Supplementary Fig. 11). APT20TTMG has not changed the levels of pro-inflammatory cytokines interferon-gamma (IFN-γ), tumor necrosis factor-alpha (TNF-α), interleukin-1beta (IL-1β), and interleukin-6 (IL-6) evaluated at 3- and 6-weeks post-surgery when compared to the vehicle group (Supplementary Fig. 12a-d).

These results suggest that APT20TTMG has a physiological effect on neuronal repair through functional remodeling of astrocytes, without inducing neuroinflammatory responses in SAMP8 mice.

### APT20TTMG does not alter NF-L levels

AD-associated neurodegeneration was investigated through neurofilament light chain (NF-L) levels measured in blood samples collected at three time-points. Recent findings indicate that blood NF-L holds great potential as a biomarker for monitoring AD. As expected, at baseline, NF-L levels were similar in treated and vehicle groups (Supplementary Fig. 12e). The same was observed at 3 weeks after surgery and at the end of treatment (Supplementary Fig. 12f, g, respectively). Therefore, the dose of 0.3 µg/day of APT20TTMG did not induce neurodegeneration at any time-point.

## Discussion

In the present study, we propose a novel therapeutic mechanism to guarantee correct splicing and suppress premature polyadenylation in AD, reducing abnormal proteins. For the first time, we show the enormous potential therapeutic effect arising from correcting the U1 snRNP complex assembly, by employing APT20TTMG treatment in relevant models, including iPSC-derived neurons from a patient diagnosed with AD and the SAMP8 mice. Notably, SAMP8 mice present a naturally accelerated aging phenotype, with classical AD hallmarks, including increased Aβ aggregates and deposition, hyperphosphorylated TAU, neuroinflammation, and cognitive deficit^12,13^.

APT20TTMG binds to U1-70K and U1-C proteins, indicating an interaction with a functional U1 snRNP complex, containing Sm proteins (not evaluated here). This is supported by studies showing that U1-C does not bind to free U1 snRNA but requires prior binding of Sm and U1-70K proteins^9,14^. The binding to U1-70K also provides evidence for the probable Sm core assembly within the immunoprecipitated pool, as U1-70K is responsible for bridging pre-U1 and U1 snRNA to the SMN complex, which mediates Sm core assembly^15^. The assembled Sm core plays an important role in the stable binding of U1-70K to the U1 snRNA^14^. All these data suggest the correct assembly of the initial machinery of splicing, indicating a subsequent spliceosome recruitment. The absence of binding to U1-A was expected, since APT20TTMG has a predicted interaction with the stem-loop II of U1 snRNA near the U1-A binding site, probably causing a low affinity of U1 snRNA to U1-A snRNP. U1-A-free molecules can bind to a conserved region of the 3’ UTR of U1-A pre-mRNA to inhibit polyadenylation^16^. Therefore, this is one of the mechanisms by which U1 snRNP safeguards pre-mRNA transcripts against premature polyadenylation and contributes to the regulation of alternative polyadenylation^17^.

Both U1-70 K and U1-A proteins have been found elevated in *postmortem* AD brains, forming aggregates in cytoplasm and tangle-like structures in neurons^7,18^. While no studies have investigated the levels of U1-70 K in SAMP8 mice, it was found that U1-70 K levels were higher in the hippocampus of non-treated animals compared to treated animals. Regarding TAU, APT20TTMG not only decreases it in AD neurons but also does not affect it in healthy neurons, which is quite significant given the fundamental roles of TAU in the physiological functioning of cells^19^. In animals, APT20TTMG also leads to a reduction in insoluble pTAU, without affecting soluble TAU. At 20 weeks old, SAMP8 mice do not exhibit neuritic plaques despite increased Aβ deposition^13,20^. However, Aβ can still form small aggregates known as *punctae*, representing an early stage of its aggregation within cells^21^. Indeed, Aβ was identified as *punctae* along cortical and hippocampal samples, with a significant reduction in its immunofluorescence in the cortex following treatment. This suggests that with a longer treatment, APT20TTMG may potentially influence the prevention of neuritic plaques. Unfortunately, extending the treatment beyond 42 days was not feasible due to the storage capacity of the infusion pump and to avoid the stress that a pump replacement would cause to the animals.

These results are supported by a study showing that U1-70 K insoluble form is strongly correlated with insoluble TAU and Aβ^11^. It is also shown that the mislocalized U1-70 K aggregate overlaps with NFTs but is already present when NFTs are not yet noticeable^22^ and that RNA splicing defects caused by U1 snRNP proteinopathy synergize with the amyloid cascade, potentially exacerbating AD progression^4^. Tissue-specific differences in TAU and Aβ were expected as insoluble TAU is predominantly found in regions associated with memory and learning, such as the hippocampus, while Aβ pathology primarily affects cortical regions^23–25^. Although targeted correction of U1 snRNP assembly did not change the cognition of the animals, an improvement would be expected if a longer treatment was feasible. These findings underscore the involvement of U1 snRNP complex dysfunction in AD and place it as an upstream event that may significantly contribute to the initial development of AD. They also evidence an enormous potential of APT20TTMG to act in earlier stages of the disease, preventing the excessive formation of NFTs by acting on pathways that precede their formation.

TAU is highly associated with the progressive mitochondrial dysfunction observed in AD^26^. APT20TTMG did not lead to mitochondrial dysfunction in iPSC-derived healthy neurons or microglia. This safe effect was also observed in the glutamate analysis. Corroborating studies that indicate a role for glutamate excitotoxicity in slowly evolving neurodegeneration, we show that glutamate release in AD neurons is higher than in healthy neurons^27^. Notably, treatment did not change extracellular glutamate levels in healthy neurons, indicating once again its safety.

In AD, axonal transport is impaired even before the appearance of classical biomarkers and morphological changes in cells are typical neuropathological hallmarks^28^. APT20TTMG did not decrease cell number and showed an upward trend in the fiber breadth of all iPSC-derived neurons. AD neurons exhibited a similar profile to non-treated healthy neurons, raising the hypothesis that APT20TTMG may improve axonal transport. There was also a non-significant increase in axonal branching in healthy neurons, treated with concentrations of 18.52 and 56.66 nM, and in AD neurons, treated with 18.52 nM. Since a reduction in the number of branches has a direct impact on regeneration and neuronal growth rate^29,30^, APT20TTMG could potentially protect neurons from degeneration. In AD, morphological alterations decrease synaptic transmission^31^ and, indeed, the electrical activity of AD neurons was not only lower than healthy neurons but also exhibited a delayed onset. Although the highest concentration tended to decrease the electrical activity of all neurons, the lowest concentration tended to increase it. In addition to the observation that neurons respond differently to the molecule, APT20TTMG, at lower concentrations than 500 nM, is not toxic to long-term neuronal cultures.

DEGs of AD neurons treated with 18.52 nM are associated with different biological processes, such as neurogenesis, neuron differentiation, cell morphogenesis, and regulation of synaptic assembly. The enrichment of these biological processes is also observed in non-treated healthy neurons when compared to non-treated AD neurons, indicating that APT20TTMG impacts the main biological processes affected by AD.

Metabolic pathway enrichment, network, and clustering analysis of DEGs of AD neurons treated with APT20TTMG revealed four clusters. In cluster I, we observed an upregulation of PI3K-Akt signaling pathway genes involved in DNA repair mechanism, microglial responses, and neuronal survival, including BRCA1, SYK, NFT3, and NTRK1^32–34^, and downregulation of pro-apoptotic and neuroinflammatory genes, such as BCL2L11, FOXO3, and JAK1^32,35^. Furthermore, the downregulation of FOXO3 could reduce Aβ levels, as it increases the activity of APP promoter and neurotoxic Aβ processing^32^. Similarly, in the cell cycle pathway, we observed the upregulation of the cyclin-dependent kinase inhibitor CDKN1A/p21 and the mediator of growth arrest GADD45A^22^, and the downregulation of the proto-oncogene MYC. The activation of the cellular senescence pathway, also in this cluster, and the downregulation of FOXO3 and MYC, combined with the upregulation of CDKN1A and GADD45A, which are also involved in this pathway, are intrinsically related to cell cycle arrest and might drive cells towards a non-proliferative state, reversing cell cycle re-entry and safeguarding neurons against apoptosis.

In cluster II, one of the key genes involved in the steroid biosynthesis, DHCR24, was upregulated and affected other genes involved in this pathway and typically downregulated in AD, such as LSS and TM7SF2, which could mitigate some of AD hallmarks^36,37^. HMGCS1, involved in the synthesis of a key intermediate in the production of ketone bodies, and ACAT2, involved in cholesterol synthesis, were upregulated. This is a great finding, as ketogenic diets have shown beneficial effects for AD by restoring metabolic dysfunction^38^. Along with ACAT2, ACSS2, involved in the synthesis of acetyl CoA and required for brain histone acetylation, was also upregulated in the pyruvate metabolism pathway. Its expression is reduced in AD, while its upregulation improves cognition^39^.

In cluster III, we observed an upregulation of ACSL3, FASN, ACACA, SCD, FADS2, SREBF1, SEC24D, PNPLA3, ABCA1, and LIPG and the downregulation of SCD5. It suggests an increase in fatty acid synthesis, particularly of polyunsaturated fatty acids, leading to a lipidogenic environment, as the AMPK pathway is upstream of the fatty acid biosynthesis pathway and holds a central role in regulating glucose and lipid metabolism, with implications for AD^40^. It is also worth mentioning the upregulation of FASN, SREBF1, ACACA, SCD, and, especially, CD36, which contributes to Aβ clearance. None of the key PPARs, linked to the regulation of fatty acids metabolism, exhibited alterations, indicating that APT20TTMG just triggers the activation of fatty acid synthesis. The upregulation of ABCA1 and LDLR seems to have a positive impact on cholesterol metabolism, as its activation promotes Aβ clearance and ApoE lipidation^41^. The upregulation of LIPG, involved in lipid hydrolysis, has not been directly correlated with the disease. However, individuals with elevated plasma LIPG levels have a higher risk of cognitive impairment^42^. Nonetheless, the potential enhancement of fatty acid biosynthesis combined with the regulation of cholesterol metabolism, which might aid in Aβ clearance and ApoE lipidation, suggests a modulation of key hallmarks by APT20TTMG.

In cluster IV, MAPK-related genes CASP3 and MYC, which are associated with Aβ burden and neuronal death^43,44^, were downregulated by APT20TTMG. In contrast, some of the upregulated genes, (e.g. GADD45A, NTF3, PRKCG, and TGFB3), are linked to the expression of the anti-apoptotic protein BCL-2, which reduces apoptosis, Aβ production, and neuroinflammation^45–48^. The decreased activation of the MAPK signaling pathway would be related to the normalized mitochondrial activity, glutamate levels, and electrical activity of neurons. This hypothesis is supported by DEGs related to the Ras signaling pathway, as Ras proteins activate protein kinases to propagate signal transduction, and its overactivation also contributes to neurodegeneration^49^. In addition to the NTF3 and PRKCG genes of the MAPK pathway, the HTR7 gene, which is decreased in AD, was upregulated, while ANGPT2, GAB2, INSR, MET, PDGFD, and SHC3, which increase the risk of developing the disease^50–56^, were downregulated.

Although no alterations were observed for NF-L, which plays a crucial role in providing structural support to neurons^57^, we observed a beneficial modulation of increased astrogliosis. This process is triggered by some pathological conditions, including misfolded proteins that induce morphological changes in astrocytes, and is one of the main characteristics of age-related impairments in the SAMP8 model^58^. The low-grade astrocyte activation, called mild astrogliosis, is considered part of the normal physiological function as a response to routine synaptic activity or minor insults and is a key factor in the clearance of TAU and Aβ^59^. Considering that GFAP levels were increased in cortical soluble fractions and presented decreased immunofluorescence, APT20TTMG appears to restore tissue homeostasis by normalizing mild astrogliosis, which is essential for regulating AD damage. Although no differences were observed in the hippocampus, the treatment did not induce an exacerbated inflammatory response in any brain structure. This leads us to suggest that the normalization of TAU and Aβ levels by APT20TTMG may effectively control astrogliosis in SAMP8 mice. At the same time, this regulated astrogliosis may also help in the clearance of previously formed Aβ aggregates.

Exploring novel therapeutic approaches is crucial for treating patients diagnosed with AD or even preventing its development. Considering the anticipated doubling of diagnosed cases in the USA by 2060^60^, there is an urgent need for innovative solutions. Given the pivotal role of the U1 snRNP complex, further research should be directed towards understanding its impact on AD and numerous other sporadic disorders. Our findings strongly support APT20TTMG as a disruptive and multi-target approach that holds significant potential to specifically decrease classical AD hallmarks by restoring U1 snRNP assembly. We recognize that the in vitro and in vivo models used in this study may not fully reflect the complexity of human AD pathology, and that the specificity of APT20TTMG towards its intended targets requires further validation. Furthermore, the long-term effects and potential off-target interactions in human systems remain to be thoroughly investigated. Despite these limitations, the results obtained thus far present a promising path for the development of therapies based on the modulation of the U1 snRNP complex. The ability of APT20TTMG to target multiple pathways relevant to AD pathology highlights its potential as an innovative therapeutic strategy. With additional studies, we hope that this approach can not only offer therapeutic relief to millions affected by AD, but also contribute to the treatment of other complex neurodegenerative disorders.

## Methods

### Ethics approval

All animal procedures were performed according to the institutional guidelines for animal care and use (NIH Publications No. 8023, revised) and the ARRIVE guidelines for animal research. All procedures in this study complied with the Animal Care and Welfare Committee and were performed according to the Animal Welfare Act from Austria (BGBl. II Nr. 542/2020, BGBl. I Nr. 76/2020, BGBl. I Nr. 86/2018, and BGBl. I Nr. 8/2022).

### APT20TTMG preparation

APT20TTMG was designed using computer-aided design strategies, synthesized by Synbio Technologies (Monmouth Junction, USA), and stored at -20 °C. For in vitro experiments, it was reconstituted in deionized water or PBS to obtain a stock solution of 100 µM. The following batches were used: 20210413004 (protein and qRT-PCR immunoprecipitation) and 20211008005 (morphological analysis, mitochondrial activity, electrophysiology, glutamate levels, TAU levels, and RNA-seq). For in vivo experiments, APT20TTMG (batch 20220913073) was reconstituted and diluted in artificial cerebrospinal (aCSF) at a final concentration of 833.3 µg/mL (3 µg/day) or 83.3 µg/mL (0.3 µg/day).

### Cells

SK-N-SH neuroblastoma cells (European Collection of Authenticated Cell Cultures, UK) were maintained and plated in Eagle’s minimum essential medium with Earle’s Balanced Salts Solutions (Gibco, USA), supplemented with 2 mM glutamine, 1% penicillin/streptomycin, 1% Non-Essential Amino Acids solution, 1 mM sodium pyruvate, and 10% fetal bovine serum. Experiments using these cells were performed at passages three to seven. Quick-Neuron™ Excitatory - Human iPSC-derived Neurons from a healthy 74 years old female donor (Elixirgen Scientific, USA), here referred to as healthy neurons, and Quick-Neuron™ Excitatory - Human iPSC-derived Neurons from a 72 years old female donor diagnosed with AD (Elixirgen Scientific, USA), here referred to as AD neurons, were maintained in Neurobasal Medium (Gibco, USA), supplemented with glutamax and antibiotics until experiments. For image evaluation, the medium was replaced with a medium without phenol red. The iCell^®^ Microglia Alzheimer’s disease and iCell^®^ Microglia wild type (FujiFilm Cellular Dynamics, USA) were maintained in iCell^®^ Glial Basal Medium (FujiFilm Cellular Dynamics, USA).

### RNA pull-down assay

SK-N-SH cells were resuspended and lysed in 150 µl ice-cold radioimmunoprecipitation assay buffer (10 mM Tris-HCl pH 7.4, 100 mM NaCl, 2.5 mM MgCl2, and 0.5% Triton X-100) (Thermo Fisher Scientific, USA) containing EDTA-free Halt protease and phosphatase inhibitor cocktail mixture (Thermo Fisher Scientific, USA) for 20 min, at 4 °C. Cell lysate (300 µg per immunoprecipitation) was added to 0.5 mg streptavidin beads (Thermo Fisher Scientific, USA) pre-conjugated with either 90 pmol biotinylated APT20TTMG or beads only (negative control) and incubated overnight, at 4 °C. Flow-through was collected and beads washed ten times with ice-cold immunoprecipitation lysis buffer. Proteins were eluted from beads in 30 µl of 4x Laemmli Sample Buffer/2-mercaptoethanol for 5 min, at 90 °C. Following determination of total protein concentration using the bicinchoninic acid (BCA) protein assay kit (Thermo Fisher Scientific, USA), samples (5 µg of input, 2.5% of flow-through, and 50% of the eluate fraction) were separated on NuPAGE™ (Thermo Fisher Scientific, USA), electrophoresed, and transferred to polyvinylidene difluoride membranes (Thermo Fisher Scientific, USA). After blocking with 5% dried milk powder diluted in tris-buffered saline, containing 0.1% Tween 20, for 1 h at room temperature, membranes were incubated overnight, at 4 °C, with the following primary antibodies: U1-70 K (1:1000; Abcam, UK), U1-A (1:1000; Abcam, UK), U1-C (1:1000; Abcam, UK), GAPDH (1:1000; Abcam, UK), followed by incubation with HRP-conjugated secondary antibody (1:5000). Proteins were visualised with enhanced chemiluminescence reagent (Thermo Scientific, USA) and images were captured using a Jess Simple Western Imaging suite (R&D Systems, USA).

Cell lysate (100 µg per immunoprecipitation) was also added to 0.5 mg streptavidin beads (Thermo Fisher Scientific, USA) pre-conjugated with either 90 pmol biotinylated APT20TTMG or nuclease-free water (beads only; negative control) and incubated overnight, at 4 °C. RNA isolated from input (50%), flow-through (50%), and eluate (100%) samples were converted to cDNA. Immunoprecipitation of auxiliary U1 RNA and total TAU was assessed by qRT-PCR using relevant primers. GAPDH was used as an endogenous control.

### Mitochondrial activity

Mitochondrial activity was measured through the quantification of the redox potential of cells using the CyQUANT™ MTT Cell Viability Assay (Invitrogen, USA), based on the conversion of 3-(4,5-dimethylthiazol-2-yl)-2,5-diphenyltetrazolium bromide (MTT) to an insoluble formazan product. iPSC-derived neurons and microglia were plated and cultured for 6 days and treated with APT20TTMG (concentrations for neurons: 6.17, 18.52, 55.56, 166.67, and 500 nM; concentrations for microglia: 18.52 and 500 nM), for 7 days. Following treatment, the culture medium was replaced by a fresh medium containing MTT solution. Cells were maintained in the CO2 incubator (37 °C, 5% CO2) for 4 h, followed by the addition of sodium dodecyl sulphate. Contents of the different wells were homogenized, and the concentration of the colorimetric probe was determined in a microplate reader, at 540 nm.

### Glutamate levels

Glutamate levels were determined using the bioluminescent Glutamate-Glo™ Assay (Promega, USA), based on the conversion of glutamine to glutamate by glutaminase enzyme. The luminescent signal produced by the reaction is proportional to the amount of glutamate in the sample and increases until complete glutamate consumption. iPSC-derived neurons were plated and cultured for 6 days. Following treatment with APT20TTMG (6.17, 18.52, 55.56, 166.67, and 500 nM) for 7 days, samples were transferred to 96-well plates and PBS (negative control) was added for background determination. Afterward, 50 µL of the glutamate detection reagent was added to wells, and plates were shaken for 60 s and maintained at room temperature, for 60 min. The luminescence reading was performed in a microplate reader and the light signal emitted by a sample was evaluated as Relative Light Units.

### TAU levels

iPSC-derived neurons were cultured for 6 days and treated with APT20TTMG (6.17, 18.52, and 500 nM) for 7 days. After treatment, cells were lysed, as previously described, and samples were evaluated using the Human TAU SimpleStep ELISA^®^ Kit (Abcam, UK), according to manufacturer instructions. For each biological replicate, quantification curves were generated.

### Neuronal morphology

iPSC-derived neurons were cultured for 6 days in NeuroHTS™ microplates (2 × 104 cells/well) (Ananda Devices, Canada). This platform enabled a robust comparison between healthy and AD neurons. After treatment with APT20TTMG (6.17, 18.52, 55.56, 166.67, and 500 nM) for 7 days, neurons were evaluated using the NeuroHTS™ 7-factor neuronal morphological profile software (Ananda Devices, Canada). The following parameters were evaluated in three distinct regions of the microplates: In the upper compartment (soma), cell number and nuclear aggregate (data not shown); in the middle compartment (axons), thickness of axonal fiber; in the bottom compartment (axons and dendrites), neurite straightness and axonal material (data not shown), number of branches, and branch junctions.

### Electrophysiology

iPSC-derived neurons (1 × 105) were plated on CytoView MEA 48 plates (Axion Biosystems, USA). After 6 days, treatment with APT20TTMG was started and repeated 3 times a week, followed by recordings. On day one of treatment, the neuron maintenance medium was removed and replaced with the medium containing two concentrations of APT20TTMG (18.52 or 500 nM). In the following days (up to 108), there was a partial replacement of the medium and the addition of the corresponding concentrations. After treatment, cells were maintained for 4 h in an incubator (37 °C, 5% CO2) and neuronal activity was measured for 5 min. Spontaneous firing measurements were performed 2 min before and 2 min after stimulation, as follows: Total time: 1 min; breakdown of stimulation: 12 times a 5-sec stimulation repeat (12 × 5 s = 1 min); each repeat is 36,400 µs of 0.5 V stimulation followed by 4.9636 s interval (0.036400 s + 4.9636 s = 5 s). Evaluation days: 6, 8, 10, 13, 15, 17, 20, 22, 24, 27, 29, 31, 34, 38, 41, 43, 45, 47, 49, 52, 54, 56, 59, 63, 65, 67, 69, 71, 73, 75, 78, 80, 82, 85, 89, 94, 96, 99, 101, 103, 106, and 108. The mean firing rate was computed as the number of spikes observed during the 5 min period and data are plotted as mean firing rate (Hz).

### RNA-seq differential expression analysis

For RNA extraction of iPSC-derived neurons cultured for 6 days and treated with 18.52 nM APT20TTMG for 7 days, the RNeasy Micro Kit (QIAGEN, Netherlands) was used, according to manufacturer instructions. Libraries were generated from 250 ng of total RNA as follows: mRNA enrichment was performed using the NEBNext Poly(A) Magnetic Isolation Module (New England Biolabs USA). cDNA synthesis was achieved with the NEBNext RNA First Strand Synthesis and NEBNext Ultra Directional RNA Second Strand Synthesis Modules (New England Biolabs, USA). The remaining steps of library preparation were performed using the NEBNext Ultra II DNA Library Prep Kit for Illumina (New England Biolabs, USA). The libraries were normalized, pooled, denatured in 0.02 N NaOH, and neutralized using HT1 buffer. The pool was loaded at 175 pM on a NovaSeq S4 lane, using Xp protocol, and a NovaSeq 6000 (Illumina, USA) was used to sequence all sample libraries. The run was performed for 2 × 100 cycles (paired-end mode). A phiX library was used as a control and mixed with libraries at a 1% level. Base calling was performed with RTA v3. The bcl2fastq2 Conversion Software v2.20 (Illumina, USA) was used to demultiplex samples and generate FASTQ reads, which were paired with a read length of 101 to achieve 100 million reads per sample. RNA-seq was performed with three replicates and one repeat.

The quality of raw reads (FASTQ files) was assessed using FASTQC v0.11.8 combined with MultiQC. Trimming was not considered necessary. Reads were aligned to the human reference genome using STAR (v2.7.6a). The GTF annotation file of the human reference genome was downloaded from Ensembl (release 102) and raw counts were calculated using FeatureCounts (v1.6.0). Differential expression analysis to determine DEGs was performed using the DESeq2 R package. Two main comparisons were performed, each with its own set of DEGs: (i) AD neurons treated with 18.52 nM APT20TTMG compared to non-treated AD neurons; and (ii) non-treated healthy neurons compared to non-treated AD neurons. Functional enrichment analysis of DEGs (focusing on gene ontology – GO) was performed with the gprofiler2 R package, using a cutoff for term size ≥ 16 and ≤ 2000. For this analysis, all DEGs from treated AD neurons were included, as well as DEGs with an absolute log2FoldChange value exceeding four when comparing healthy to AD neurons. For network and enrichment analysis of metabolic pathways of DEGs in AD neurons treated with 18.52 nM APT20TTMG compared to non-treated AD neurons, the STRING app (v2.0.1) of Cytoscape (v3.11.1; The Cytoscape Consortium, USA) was applied using the functional enrichment option (focusing on the Kyoto Encyclopedia of Genes and Genomes – KEGG). Further visual annotation with labels of the enrichment terms names and clustering of the enrichment network was performed with the Cytoscape AutoAnnotate app (v1.4.1). Clusters comprising more than six genes and enriched pathways with the most significant false discovery rates (FDR) were prioritized.

### Animals

Female SAMP8/TaHsd mice aged 7–8 weeks were obtained from Envigo (USA). Upon arrival, the initial health status of the animals was checked, and they were then transferred to individually ventilated cages. Animals were maintained in the QPS Austria animal facility, accredited by the Association for Assessment and Accreditation of Laboratory Animal Care (AAALAC, USA). Only animals in apparently good health condition were included in the study. Randomization of group allocation was done per cage. Animals were assigned to different starting groups (cohorts) comprising animals of all treatment groups. The number of animals in a starting group was limited to ensure the same age and uniform handling.

### Pump implantation and brain infusion

For the implantation of the ALZET^®^ Mini-Osmotic Pump, model 2006 (ALZET^®^, USA), all animals underwent surgery. After anesthesia with isoflurane (induction: 4.5%; maintenance: 1–3%), buprenorphine was applied once before surgery at 0.04 mg/kg, and animals had the fur of the head and placement area of the pump shaved. They were kindly placed in a stereotaxic apparatus, on a heating pad to prevent hypothermia, and their eyes were covered with an eye ointment for protection. After disinfection, a midline incision of the scalp was made, and the skull was cleaned for visualization of suture lines. Using the mouse brain atlas of Paxinos and Franklin (2001), a hole was drilled above the target regions according to the following coordinates, measured from dura mater: anterior/posterior = -0.5 mm, midline = 1.0 mm, and dorsoventral = 1.7 mm. Also, a long incision (1 cm) was made on the side chosen for the pump placement and hemostatic forceps were inserted into the incision to generate a pocket for the pump. The pump, attached to the catheter and the brain cannula, was inserted into the subcutaneous (s.c.) pocket with the delivery port from the pump heading towards the cannula site. The cannula was fixed in the head using cyanoacrylate adhesive and the wounds were closed with a surgical suture. The exact location of the cannula implantation is shown in Supplementary Fig. 13. Carprofen was administered at 5 mg/kg via s.c. immediately after the surgery and for 2 days post-surgery. During the wake-up time, 0.9% saline was injected via s.c. Heat lamps were provided to help with the recovery of the animals. Wet food was provided for 1 week after surgery, as well as water bottles with long drinking caps and food on the ground of cages during the whole study.

APT20TTMG (83.3 µg/mL/0.3 µg per day or 833.3 µg/mL/3 µg per day) or vehicle (aCSF) were injected via intracerebroventricular (i.c.v.) using ALZET^®^ Mini-Osmotic Pump, with a flow rate of 0.15 µl/h, continuously for 42 days. After implantation of ALZET^®^ minipumps, health status was monitored three times per day for three days, followed by health monitoring once per day for an additional 11 days. Bodyweight of animals was assessed at baseline and from week one to six.

### Behavioral analysis

During the fifth week of treatment, the spatial memory of animals was evaluated in the MWM test. The test consisted of one training session, with four trials of 60 s each, during four consecutive days, and one probe trial on day five. It was performed in a circular pool (100 cm diameter and 30 cm high), filled with water (20 cm depth) at 25 ºC, divided into four quadrants (Northeast, NE; Northwest, NW; Southeast, SE; Southwest, SW). In all trials, a platform was placed in the NE quadrant, at 1 cm below the surface of the water. Artificial landmarks were mounted above the walls of the pool for animal orientation. For the training session, mice were released from predefined positions each day (Supplementary Fig. 14). Animals that did not find the hidden platform were guided to it by the experimenter and stood there for 10–15 s to orientate in the surroundings and to rest. During the probe trial, performed on day five, the platform was removed from the pool. The escape latency to find the former platform target position, distance, target zone crossings, and abidance in the target quadrant were recorded using the EthoVision XT 14 video tracking system (Noldus Information Technology, the Netherlands).

In the sixth week, emotional learning was evaluated using the CFC test. It was performed in an automated conditioning chamber (22 cm width, 22 cm length, and 25 cm height) (TSE Systems, Germany), for two days. The light in the chamber was set to 30 lx. On the training day, 5 s after being placed into the chamber, mice received a light electrical foot shock of 0.5 mA for 2 s, and 30 s later they were returned to their home cage. One day later, they were placed again in the conditioning chamber for 5 min, without any shock, and freezing behavior was analyzed in 1-min intervals. On both days, animals were recorded by the automated chamber system.

### Blood collection

In vivo blood was collected by submandibular bleeding from the facial vein/artery plexus, without anesthesia, at baseline and 3 weeks after surgery. Maximum allowed blood volume was sampled into MiniCollect^®^ K2EDTA tubes, which were inverted thoroughly, and centrifuged at 3.000 × g for 10 min, at room temperature (22 °C). Plasma was transferred to a pre-labeled LoBind^®^ Tube (Eppendorf, Germany) and frozen on dry ice. For terminal blood collection, mice were terminally anesthetized by intraperitoneal injection of pentobarbital (600 mg/kg, dosing 10 µl/g body weight), 42 days after implantation of pumps. Their thorax was opened, and the blood collected by heart puncture with a 23-gauge needle, then processed as previously described. Blood samples were stored at -80 °C.

### Neurofilament-light chain measurement

NF-L levels were analyzed in both in vivo and terminal plasma using the NF-light^®^ ELISA (UmanDiagnostics, Sweden), according to manufacturer instructions. Briefly, samples (100 µl) were added to the pre-coated wells and incubated for 1.5 h under constant agitation (800 rpm), at room temperature. Then, they were incubated with 100 µl of the tracer antibody for 45 min, 100 µl of the conjugated antibody for 30 min, and 100 µl of TMB substrate for 15 min, with three consecutive washings with Wash Buffer between each incubation. After adding 50 µl of the stop reagent, the plate was read at 450 nm on the Cytation 5 Cell Imaging Multimode Reader (Biotek, USA). Data were evaluated in comparison to calibration curves provided in the kit. Results are expressed as pg/ml plasma.

### Cytokines measurements

The V-PLEX Human Proinflammatory Panel I (4-Plex) (Meso Scale Discovery, USA) was used to measure IFN-γ, TNF-α, IL-1β, and IL-6 in plasma samples collected at 3 and 6 weeks after surgery, according to manufacturer instructions. Measurements were conducted on the MESO QuickPlex SQ 120 (Meso Scale Discovery, USA) and the levels were evaluated in comparison to calibration curves provided in the kits. Results are expressed as pg/ml plasma.

### Brain collection and fractionation

Brains were collected after transcardial perfusion with ice-cold PBS. A constant pressure of 100 to 120 mm Hg was maintained on the perfusion solution by connecting the solution bottle to a manometer-controlled air compressor. The skull of animals was opened, and brains were removed and hemisected on a cooled surface. The left hemispheres were dissected into cortex and hippocampus and snapped frozen for biochemical analysis. The right hemispheres were processed and embedded in cryomolds for immunohistochemical analysis. The soluble and insoluble brain fractionation was performed according to Julien, Bretteville, and Planel (2012)^61^. After homogenization in cold H buffer (10 mM Tris, 1 mM EGTA, 0.8 M NaCl, 10% sucrose, pH 7.4, containing 1 mM phenylmethylsulfonyl fluoride), samples were centrifuged twice at 20,817 × g during 42 min, at 4 °C. The supernatant collected after both centrifugations were combined, added to 1% sarkosyl, incubated on an orbital shaker (700 rpm) for 1 h, at 37 °C, and centrifuged at 150,000 × g for 35 min, at room temperature. The resulting supernatant (soluble fraction) was collected and stored at -80 °C. The pellet (insoluble fraction) was resuspended in TBS (10 mM Tris and 154 mM NaCl) and stored at -80 °C. The BCA protein assay kit (Thermo Scientific, USA) was used to determine protein concentration, according to manufacturer instructions.

### Total TAU, pTAU231, and GFAP

Total TAU and phosphorylated TAU at residue Thr231 (pTAU231) were measured in both soluble and insoluble fractions from cortical and hippocampal samples, using the Phospho (Thr231)/Total TAU Kit (Meso Scale Discovery, USA), according to manufacturer instructions. GFAP levels were measured in the soluble fraction from cortical and hippocampal samples using the R-PLEX Human GFAP Assay (Meso Scale Discovery, USA), according to manufacturer instructions. All measurements were conducted on the MESO QuickPlex SQ 120 (Meso Scale Discovery, USA) and the levels were evaluated in comparison to calibration curves provided in the kits. Results are expressed as pg/µg total protein, for total TAU and GFAP, and arbitrary units (AU), for pTAU231.

### Immunohistochemistry

For fixation, right brain hemispheres were immersed in freshly prepared ice-cold 4% paraformaldehyde diluted in PBS, pH 7.4, at 4 °C. After 48 h, they were transferred to a 15% sucrose diluted in PBS for 24 h, at 4 °C. Tissue blocks were transferred to cryomolds, embedded in optimal cutting temperature compound (Sakura Finetek USA), frozen in dry ice-cooled isopentane and stored at -80 °C. Five consecutive cryosections were sagittally cut at 10 μm thickness on a Leica CM1950 (Leica Biosystems, Germany) or Cryostar NX70 (Thermo Scientific, USA) cryostats. A collection scheme was repeated for 12 sectioning levels, chosen according to the mouse brain atlas of Paxinos and Franklin (2001) (Supplementary Fig. 13). Collection of cryosections started at a level around 0.2 mm lateral from the midline and extended through the hemisphere, to ensure systematic random sampling through the target regions. A total of 60 cryosections per animal was collected and stored at -20 °C.

Cryosections were air-dried for 45 min, washed in PBS for 10 min, and incubated with M.O.M.^®^ (Mouse on Mouse) Blocking Reagent (Vector Laboratories, USA), diluted in 0.1% Triton X-100 in PBS, in a damp chamber for 60 min, at room temperature. After three consecutive 5-min washing with PBS, they were incubated overnight, at 4 °C, with the following primary antibodies in M.O.M.^®^ diluent (Vector Laboratories, USA): mouse anti-beta-Amyloid (17–24) [4G8] (1:5000; Biozym Scientific GmbH, Germany), mouse anti-phosphorylated TAU (pSer202/Thr205) [AT8] (1:300; Thermo Scientific, USA), rabbit anti-glial fibrillary acidic protein (GFAP) (1:2000; Dako, USA), and rabbit anti-U1-70 K (1:100; Sigma-Aldrich, USA). For anti-phosphorylated TAU (pSer202/Thr205) and anti-U1-70 K, after being air-dried, cryosections were incubated with 10% citrate buffer (LabVision, Sweden) for 15 min at 95 °C, in a steamer, and then for 15 min at room temperature. After washes, as previously described, cryosections were incubated for 60 min, at room temperature, with the following secondary antibodies in M.O.M.^®^ diluent, protected from light: donkey anti-guinea pig Cy™3 (1:500; Jackson ImmunoResearch, USA), donkey anti-mouse DyLight™ 550 (1:500; Thermo Scientific, USA), donkey anti-mouse DyLight™ 650 (1:500; Thermo Scientific, USA), and donkey anti-rabbit Alexa Fluor^®^ 750 (1:500; Abcam, USA), donkey anti-rabbit DyLight^®^ 650 (1:500; Abcam, USA). Cryosections were washed, as previously described, incubated with DAPI working solution for 15 min, and washed twice with PBS and once with double-distilled water, each for 5 min. Cryosections were automatically covered with Mowiol (Sigma-Aldrich, USA) and coverslips using a Leica CV5030 cover slipper (Leica Biosystems, Germany).

Whole slide scans of the stained cryosections were recorded on a ZEISS automatic microscope Axio Scan.Z1 (ZEISS, Germany), equipped with a ZEISS Axiocam 506 mono (ZEISS, Germany) and an HV-F202SCL camera (Hitachi Kokusai America, USA). The ZEISS ZEN 3.3 software (ZEISS, Germany) was used for slide scan acquisitions, whereas Image Pro 10 (Media Cybernetics, USA) was used for image analysis. Target areas were identified by drawing regions of interest (ROI) and immunoreactive objects were detected by adequate thresholding and morphological filtering. The following object features were quantified: immunoreactive area (percentage of cumulative object area based on ROI size), size of above-threshold objects, mean object intensity (mean signal intensity of identified objects) and mean object density (number of objects normalized to ROI size).

### Statistics

Normally distributed data obtained from two treatment groups were analyzed by Student’s t-test. For comparisons between three groups with normally distributed data, one-way ANOVA, followed by Tukey’s or Dunnett’s post hoc test, was performed. Neuronal morphology was evaluated using the Kruskal-Wallis nonparametric test, followed by Dunn’s post hoc test. When data did not present a normal distribution, the Mann-Whitney test and the Kruskal-Wallis test, followed by Dunn’s post hoc test, were performed for comparisons between two or three treatment groups, respectively. Longitudinal analyses of data were performed using two-way ANOVA, followed by Tukey’s multiple comparisons post hoc test. Data were analyzed in GraphPad Prism™ 10 (GraphPad Software Inc., USA) and are represented as mean ± standard error of the mean (SEM). Statistical significance was considered as *P < 0.05, **P < 0.01, ***P < 0.001, and ****P < 0.0001.

## Electronic supplementary material

Below is the link to the electronic supplementary material.

Supplementary Material 1

Supplementary Material 2

Supplementary Material 3

Supplementary Material 4

Supplementary Material 5

Supplementary Material 6

Supplementary Material 7

Supplementary Material 8

Supplementary Material 9

Supplementary Material 10

Supplementary Material 11

Supplementary Material 12

Supplementary Material 13

Supplementary Material 14

Supplementary Material 15

Supplementary Material 16

## Data Availability

The data that support the findings of this study are available from the corresponding author, upon reasonable request.
