# Supplementary figures and images for: Effects of the therapeutic correction of U1 snRNP complex on Alzheimer’s disease
Source: Sci Rep. 2024 Dec 3;14:30085. doi: 10.1038/s41598-024-81687-2 (PMC11615310; doi:10.1038/s41598-024-81687-2)

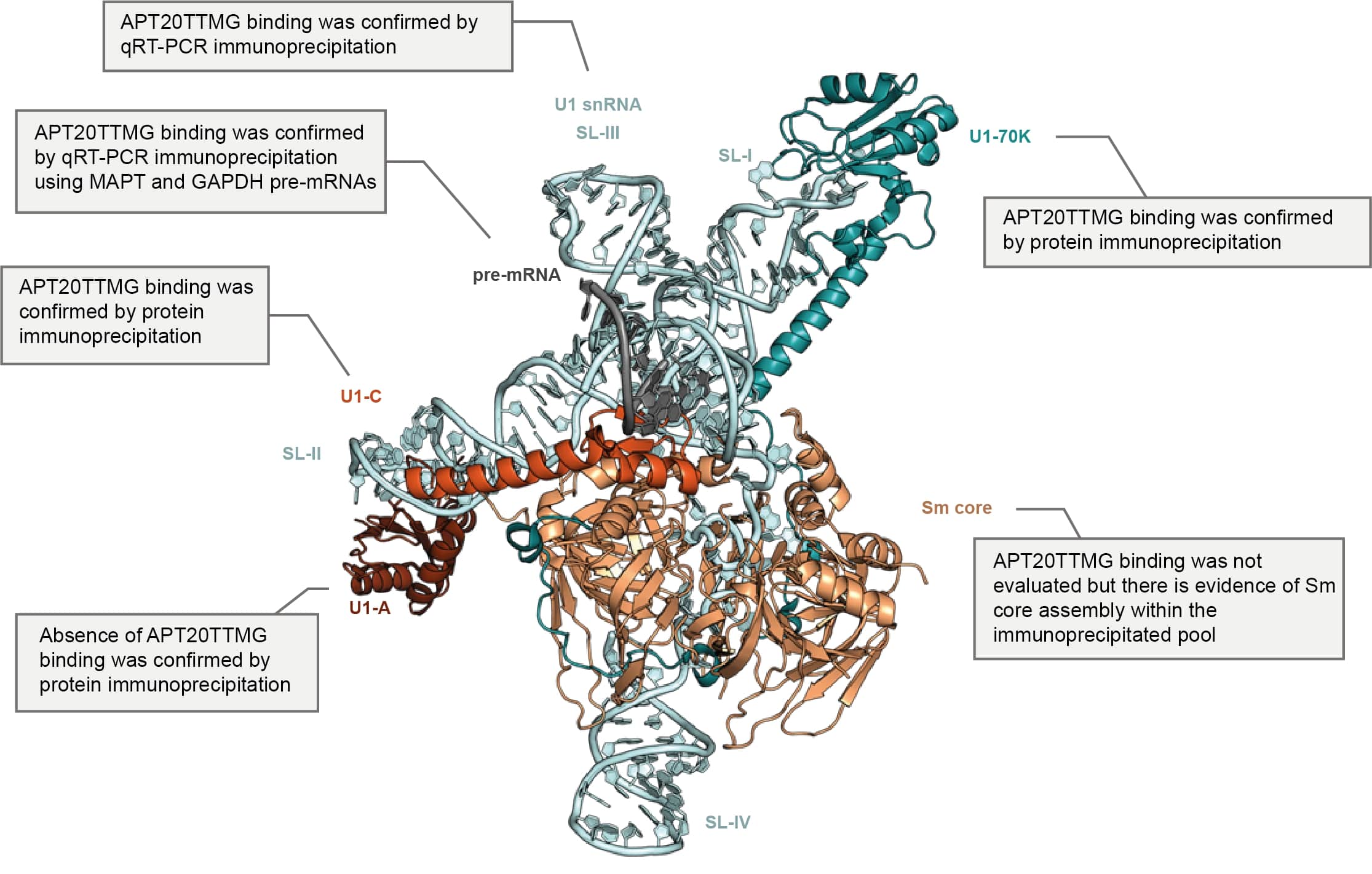

Supplement: Supplementary file 2 — Supplementary Material 2 [file 41598_2024_81687_MOESM2_ESM.jpg]

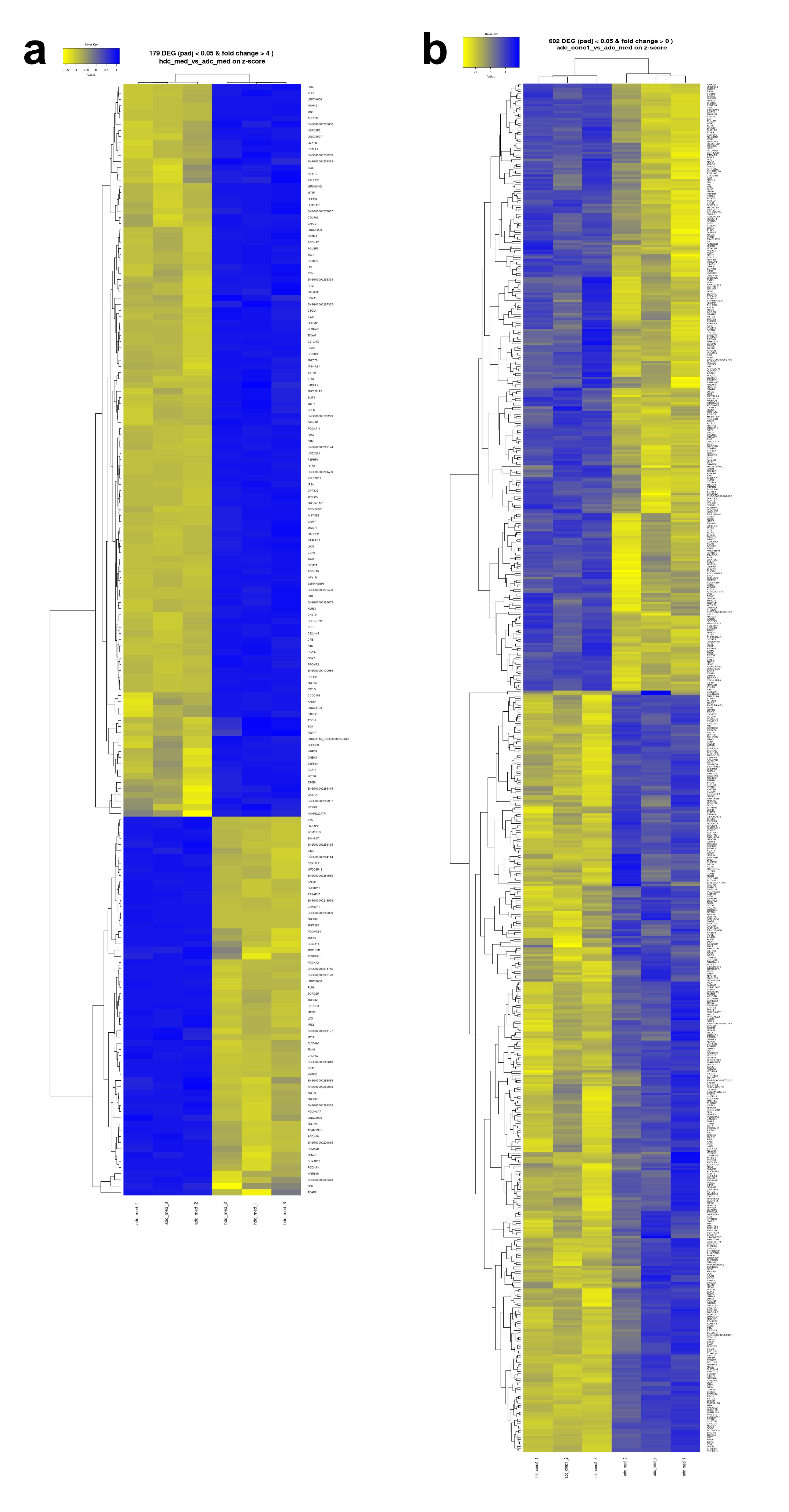

Supplement: Supplementary file 4 — Supplementary Material 4 [file 41598_2024_81687_MOESM4_ESM.jpg]

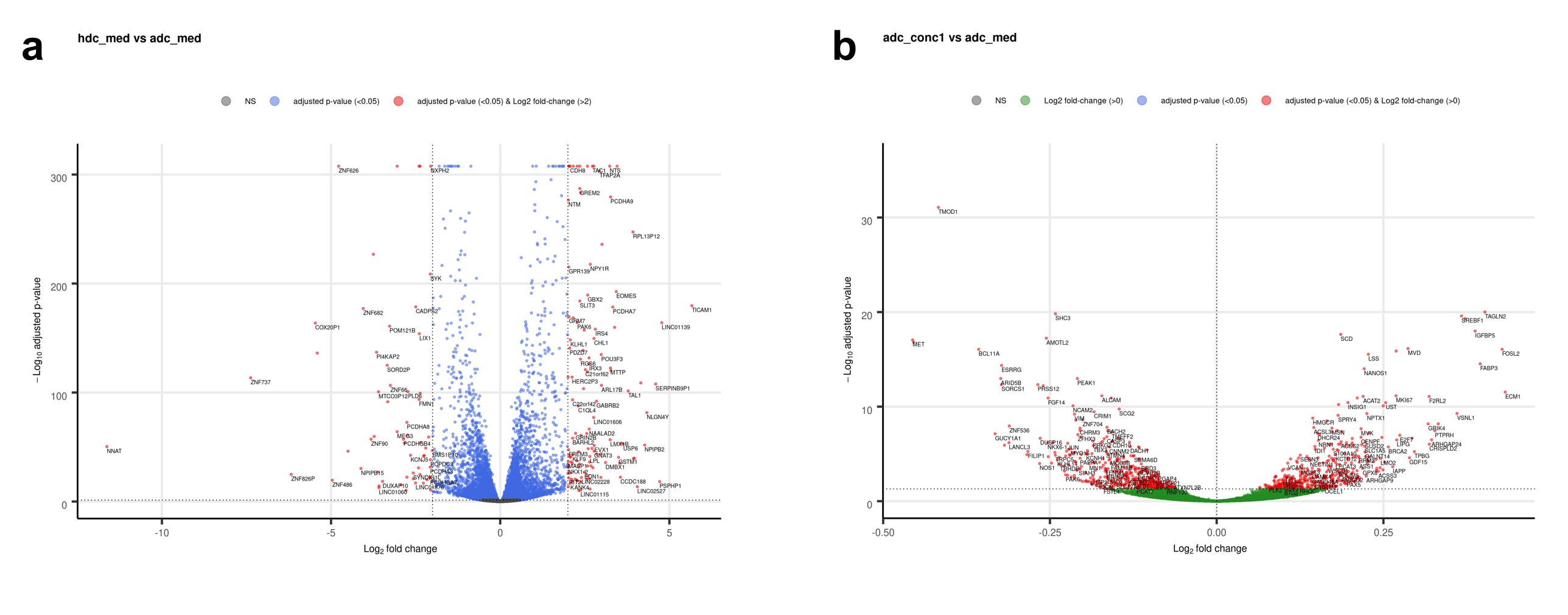

Supplement: Supplementary file 5 — Supplementary Material 5 [file 41598_2024_81687_MOESM5_ESM.png]

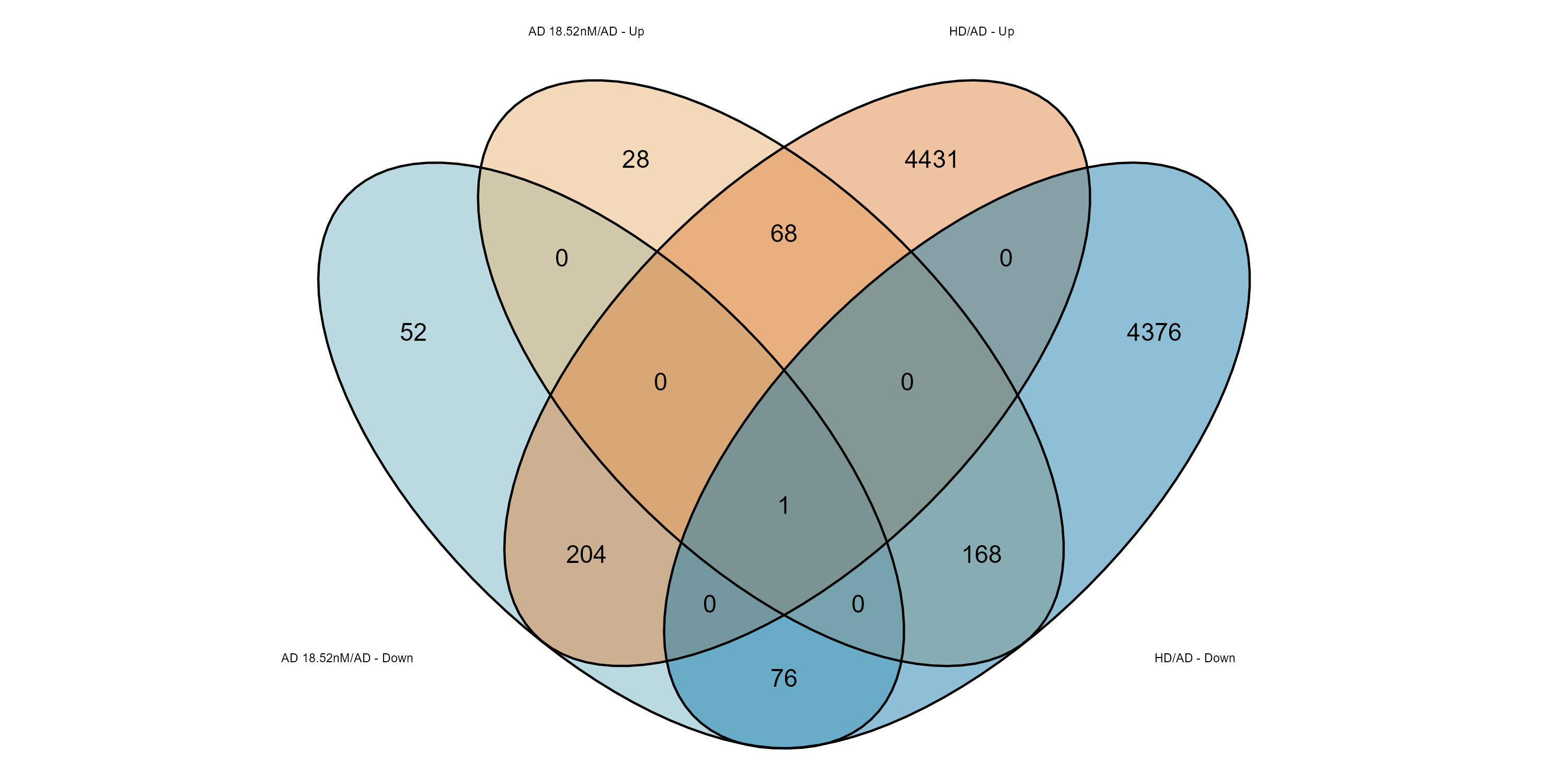

Supplement: Supplementary file 6 — Supplementary Material 6 [file 41598_2024_81687_MOESM6_ESM.png]

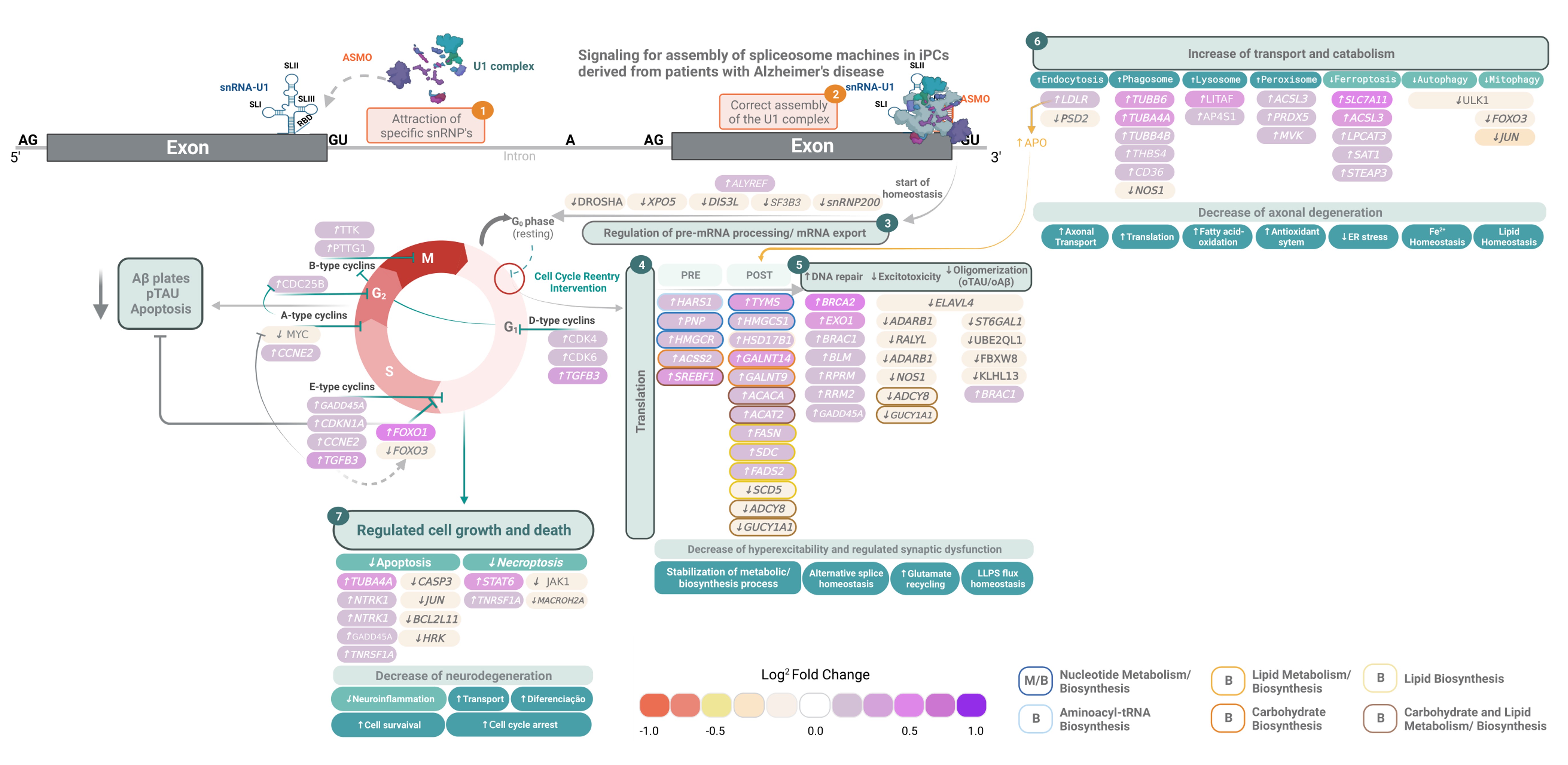

Supplement: Supplementary file 7 — Supplementary Material 7 [file 41598_2024_81687_MOESM7_ESM.jpg]

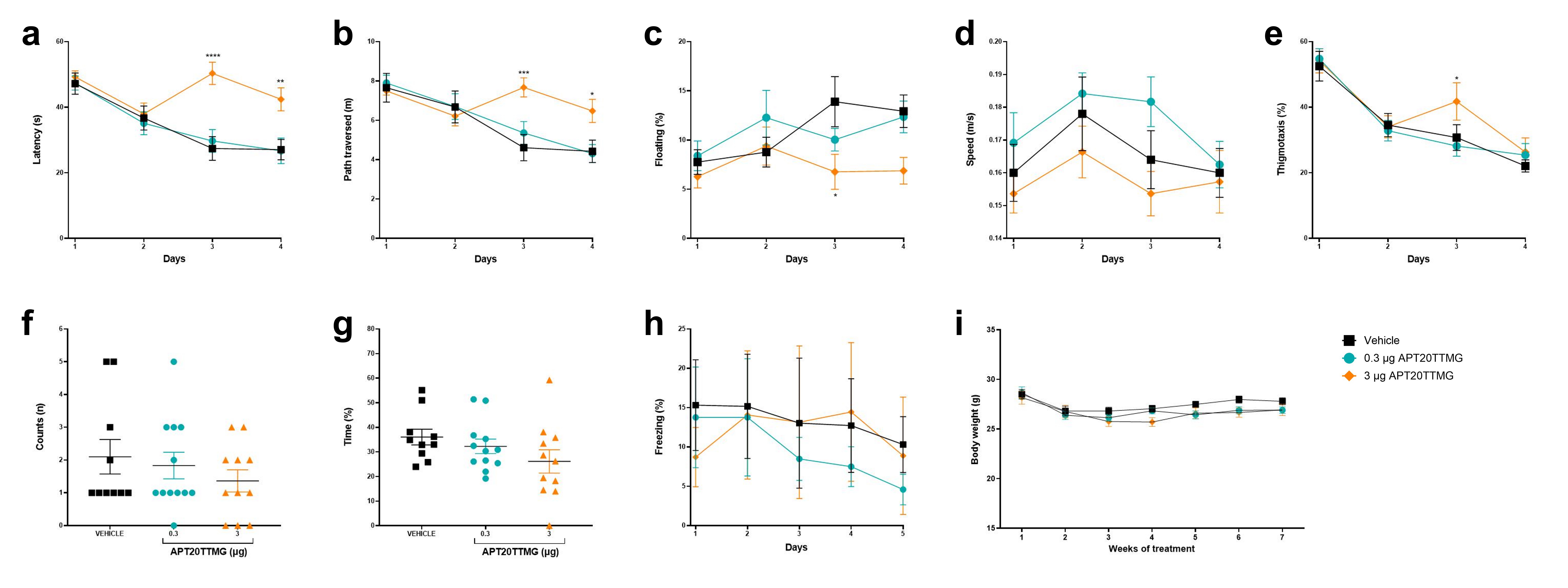

Supplement: Supplementary file 8 — Supplementary Material 8 [file 41598_2024_81687_MOESM8_ESM.jpg]

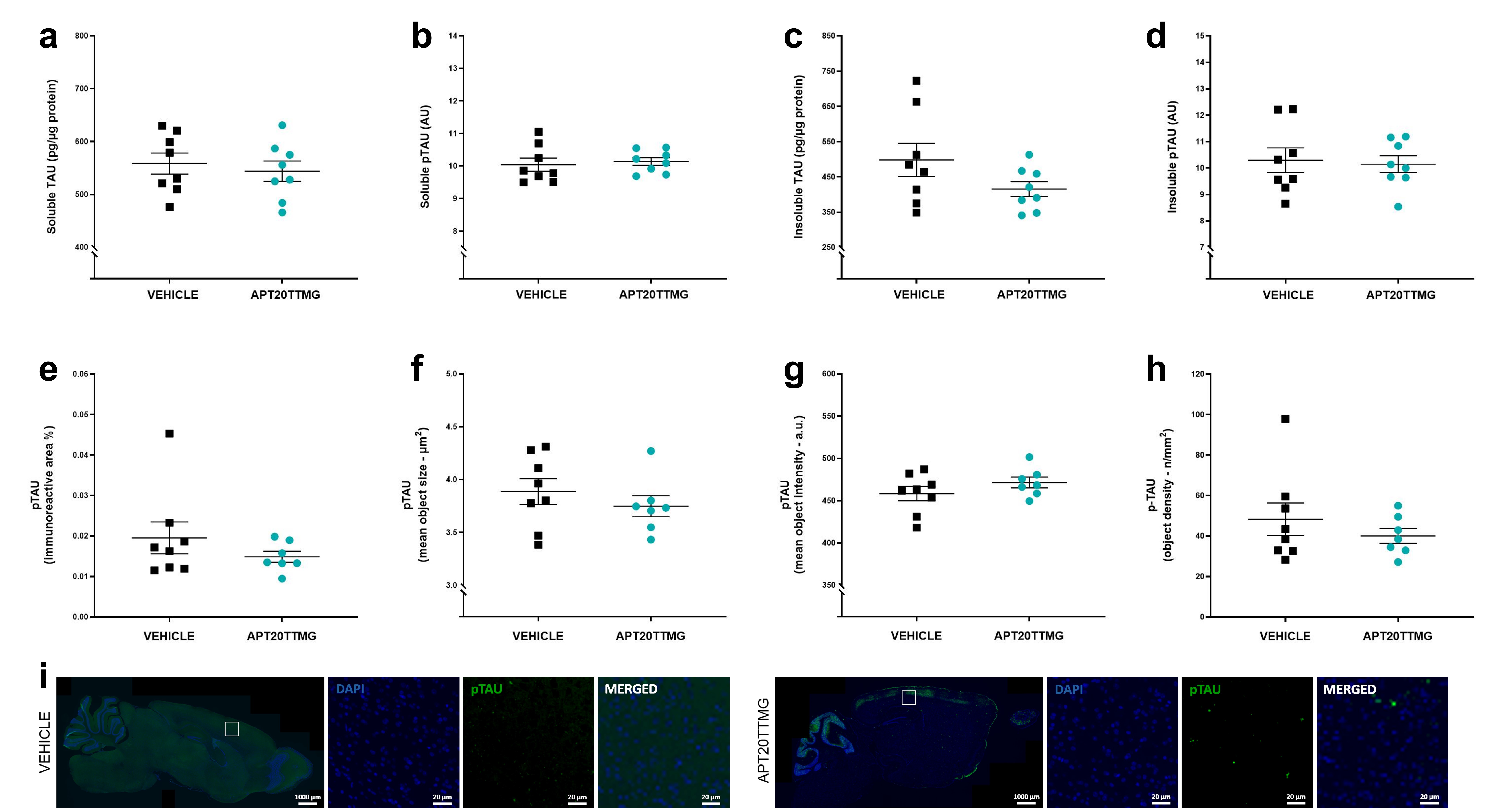

Supplement: Supplementary file 9 — Supplementary Material 9 [file 41598_2024_81687_MOESM9_ESM.jpg]

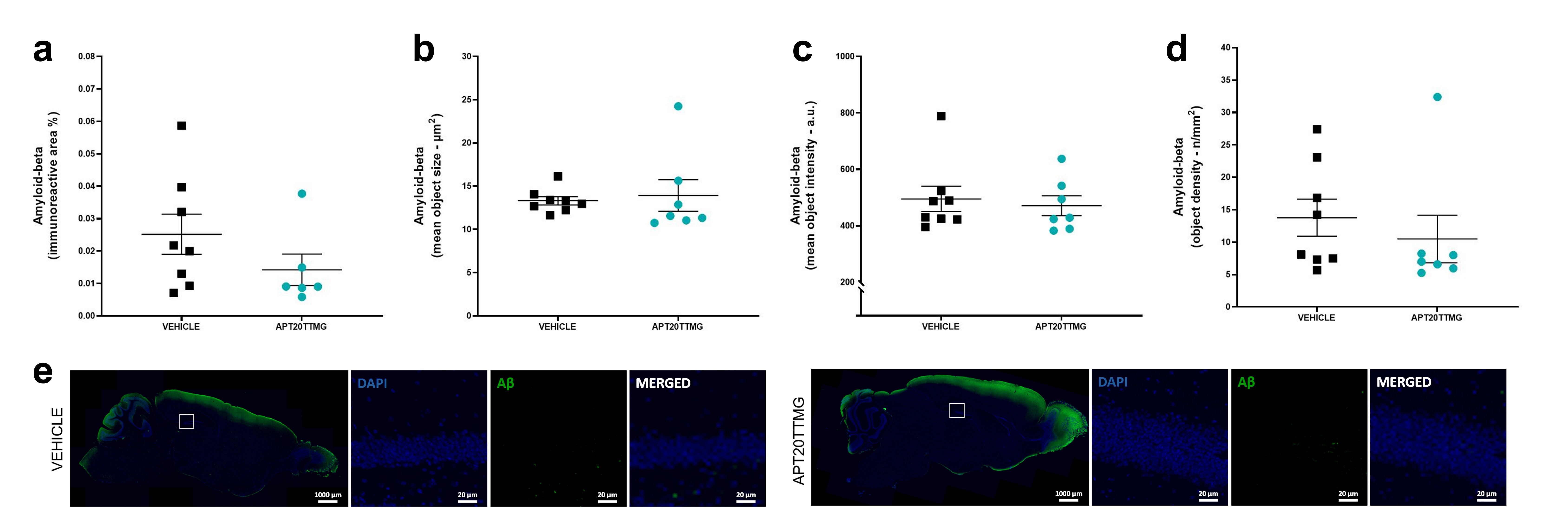

Supplement: Supplementary file 10 — Supplementary Material 10 [file 41598_2024_81687_MOESM10_ESM.jpg]

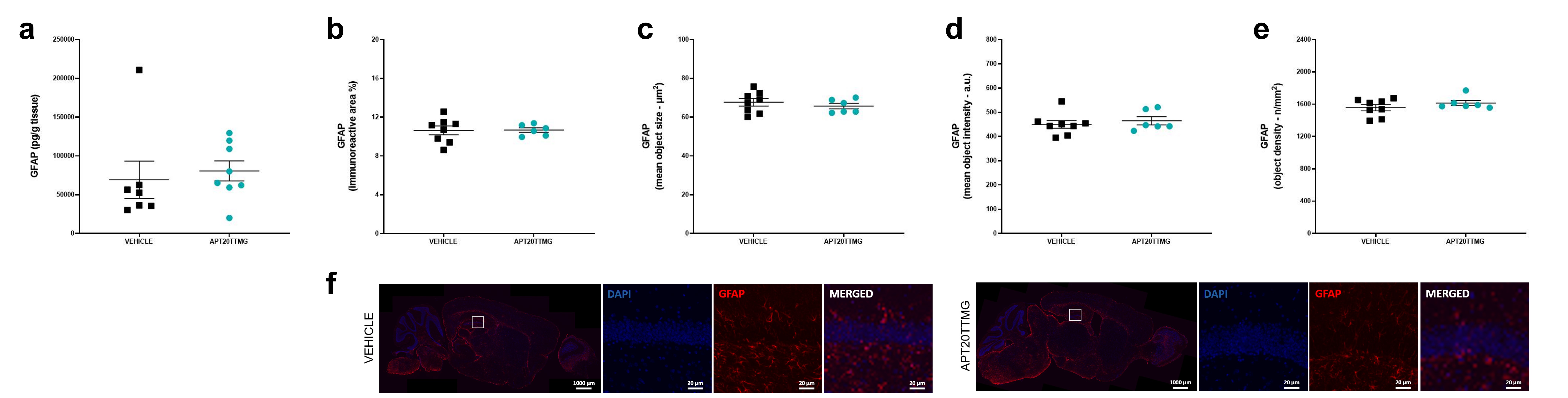

Supplement: Supplementary file 11 — Supplementary Material 11 [file 41598_2024_81687_MOESM11_ESM.jpg]

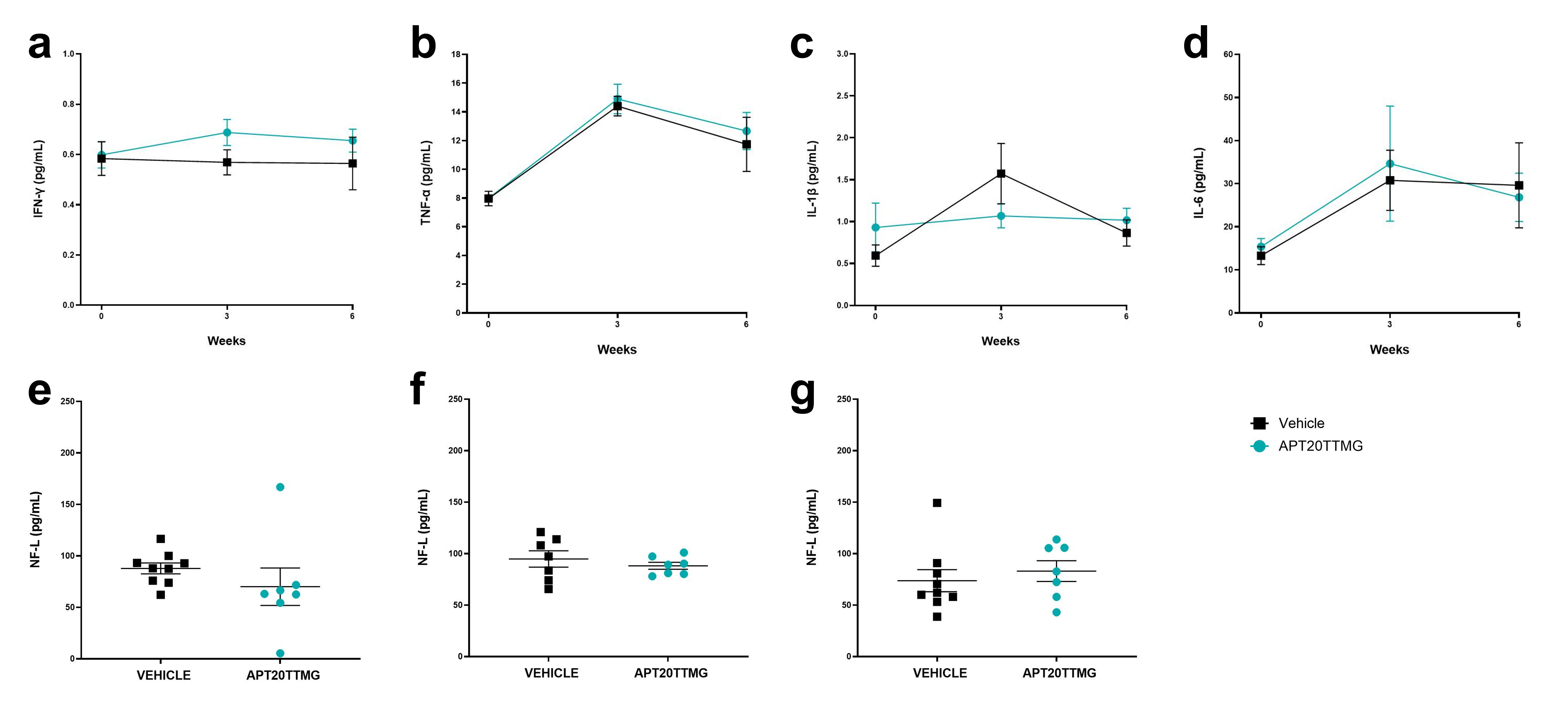

Supplement: Supplementary file 12 — Supplementary Material 12 [file 41598_2024_81687_MOESM12_ESM.jpg]

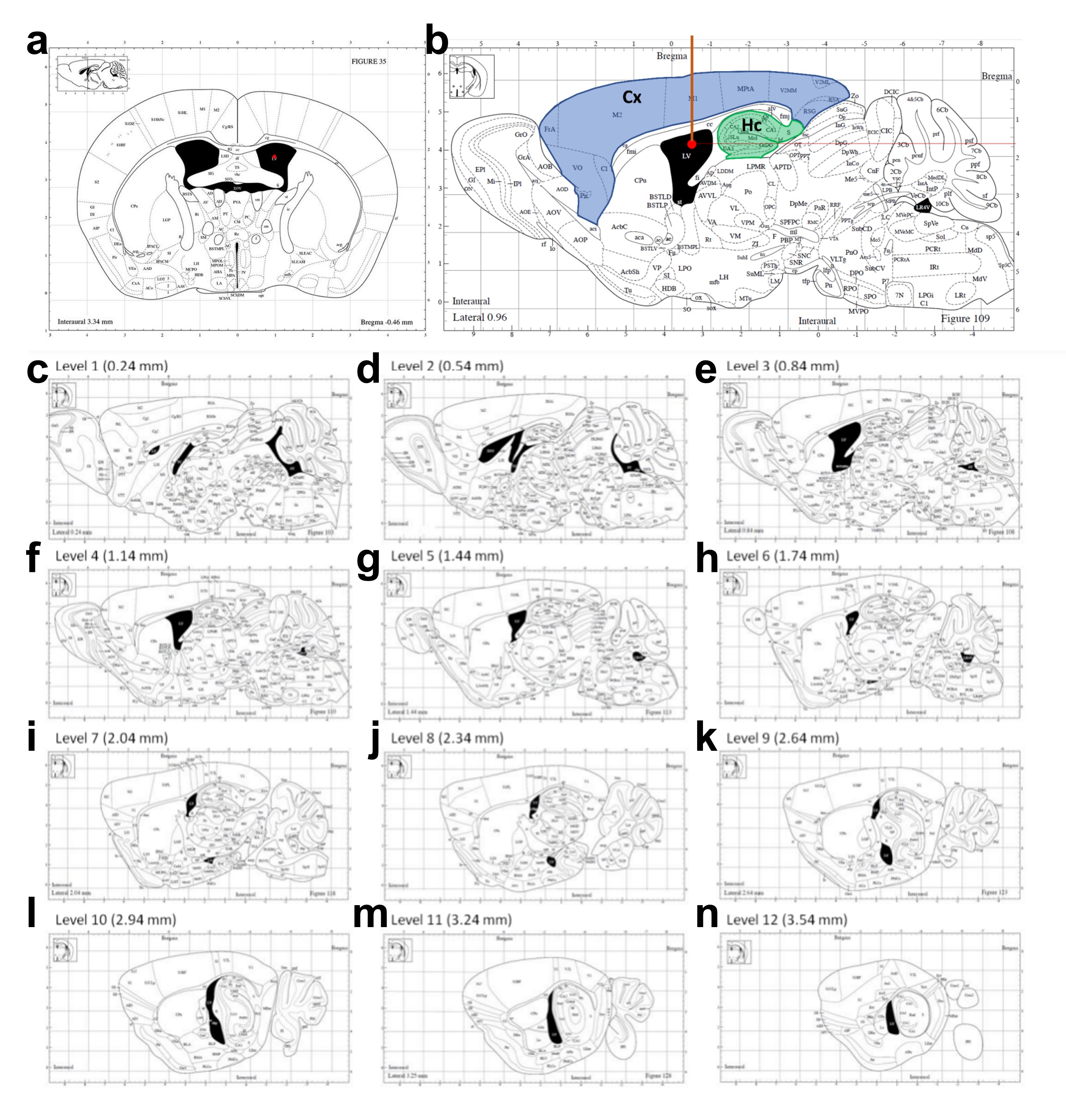

Supplement: Supplementary file 13 — Supplementary Material 13 [file 41598_2024_81687_MOESM13_ESM.jpg]

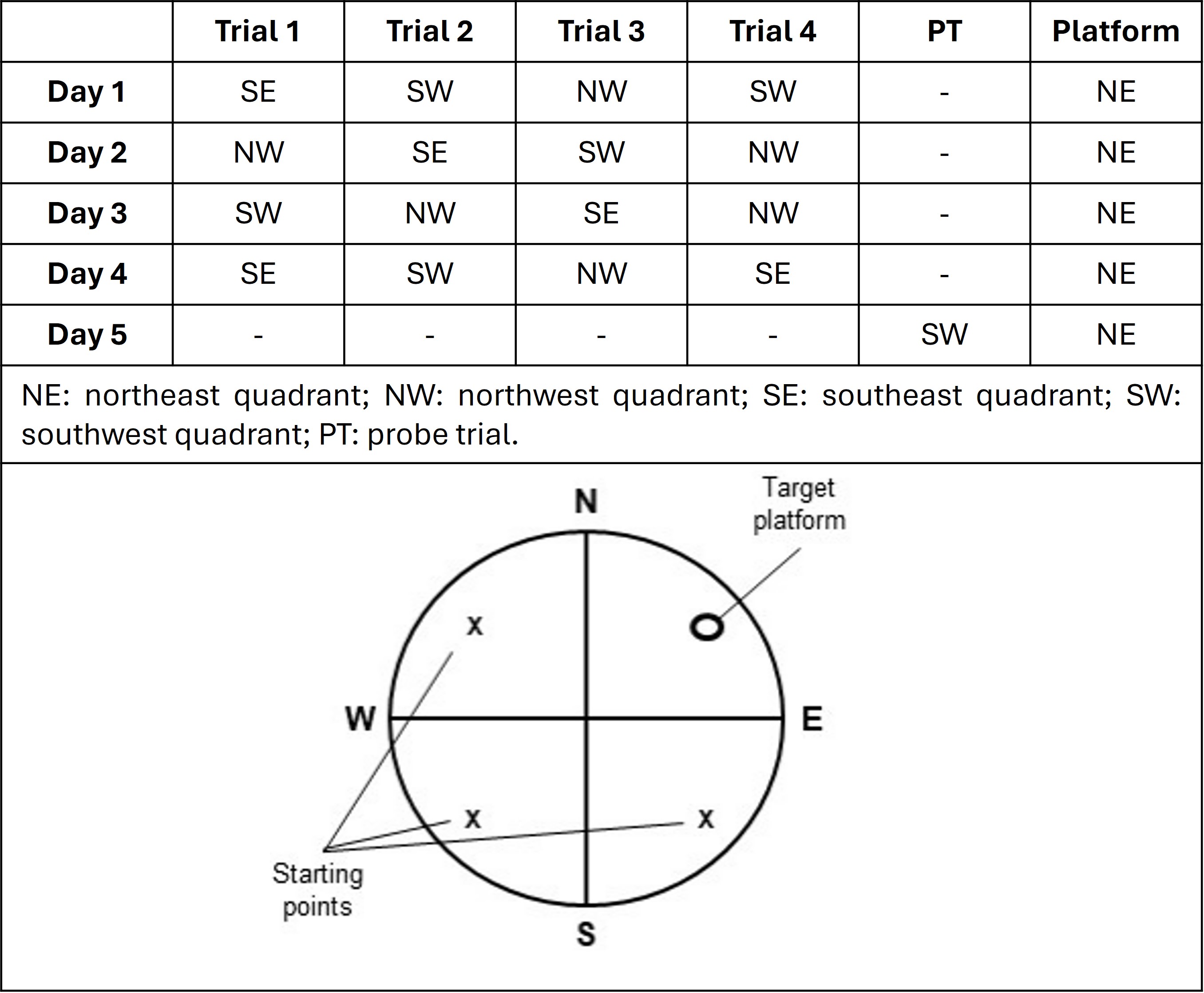

Supplement: Supplementary file 14 — Supplementary Material 14 [file 41598_2024_81687_MOESM14_ESM.jpg]

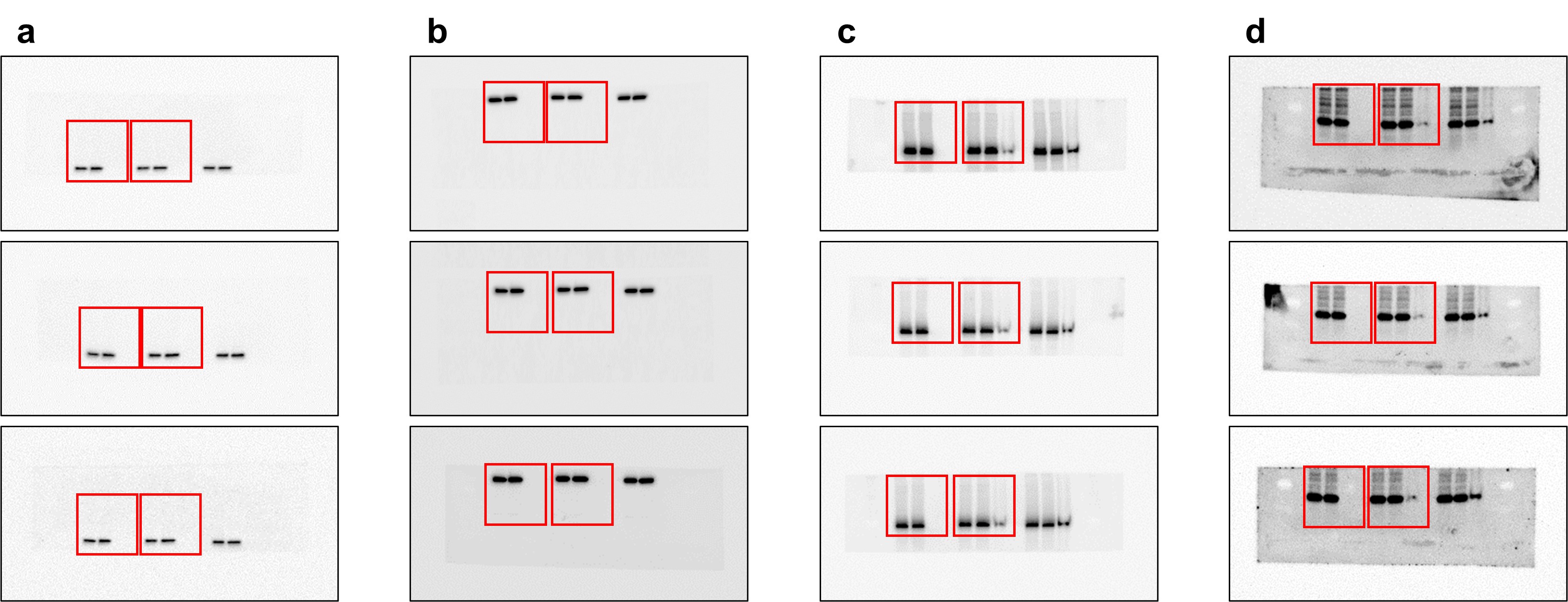

Supplement: Supplementary file 16 — Supplementary Material 16 [file 41598_2024_81687_MOESM16_ESM.jpg]
