# Supplementary material for: Effects of the therapeutic correction of U1 snRNP complex on Alzheimer’s disease
Source: Sci Rep. 2024 Dec 3;14:30085. doi: 10.1038/s41598-024-81687-2 (PMC11615310; doi:10.1038/s41598-024-81687-2)
Supplement: Supplementary file 15 — Supplementary Material 15 [file 41598_2024_81687_MOESM15_ESM.docx]

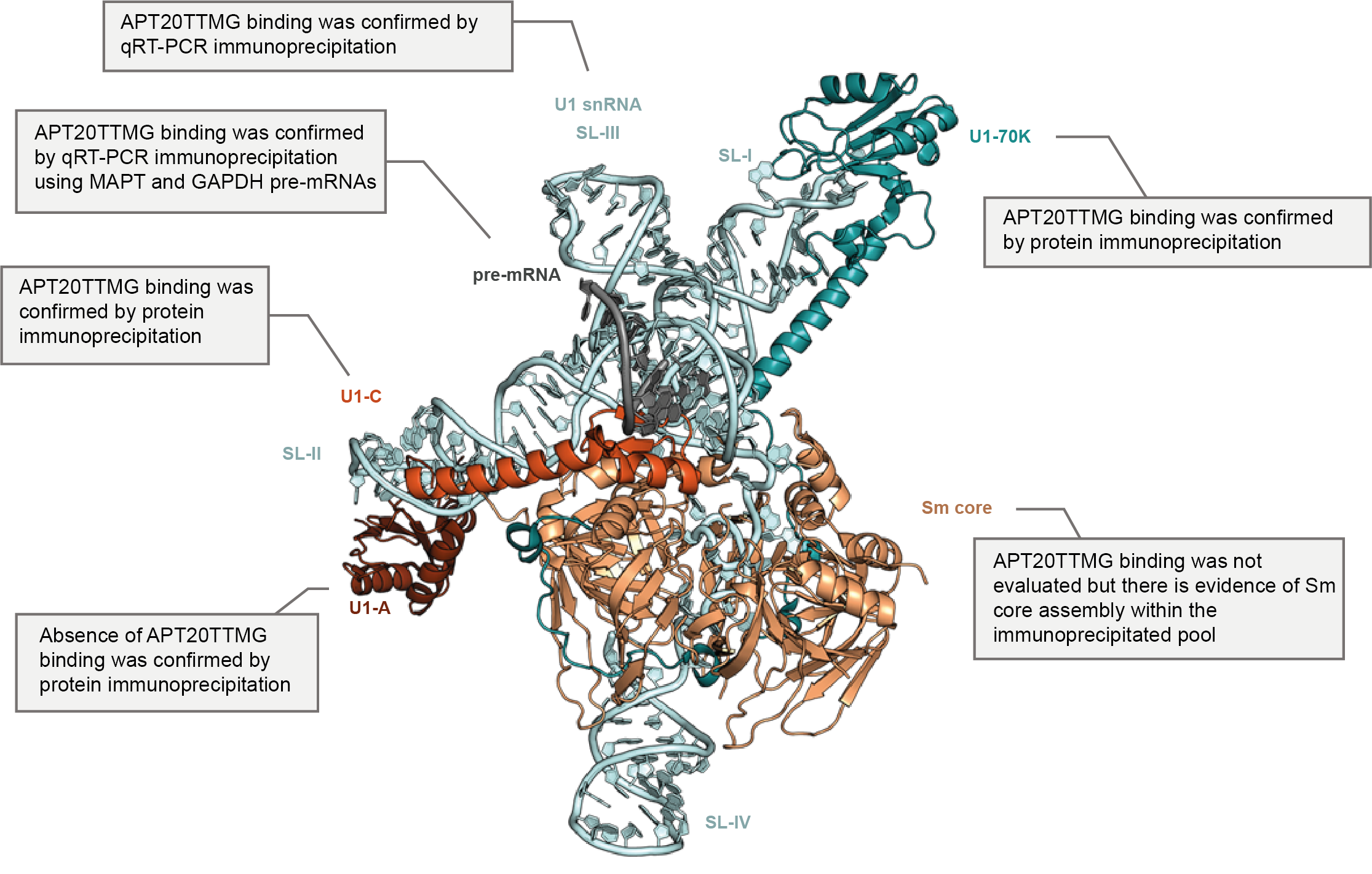


**Supplementary Fig. 1. APT20TTMG binds to key components of the U1 snRNP complex.** The U1 snRNP complex structure representation was generated using PDB 6QX9 (3.28 Å, solved with Cryo EM method) obtained from the protein data bank website and the open source PyMOL software (v2.6.0a0). The U1-C protein is represented in dark orange. U1-70K is represented in dark blue. U1 snRNA is represented in light blue. The SM ring is represented in light orange. U1-A is represented in red. The pre-mRNA fragment is represented in gray. The text in the boxes highlights the main outcomes obtained with the immunoprecipitation assays for each component of the U1 snRNP complex. SL: stem-loop.

**d**

**c**

**b**

**a**


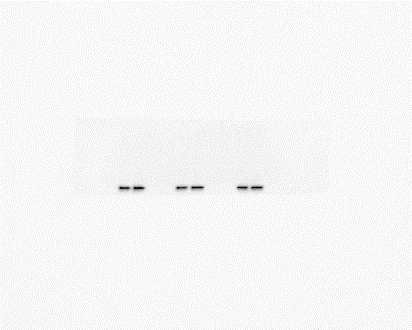

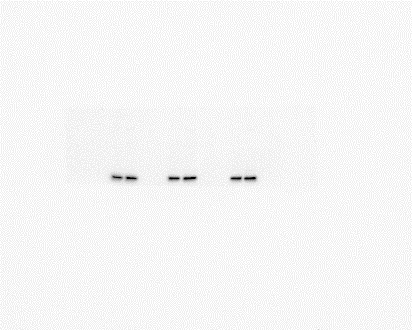

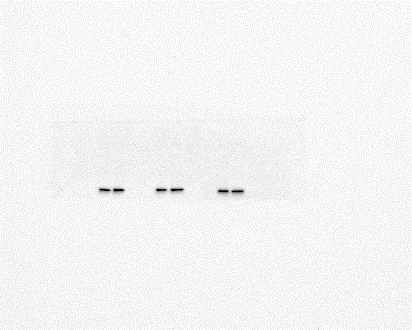

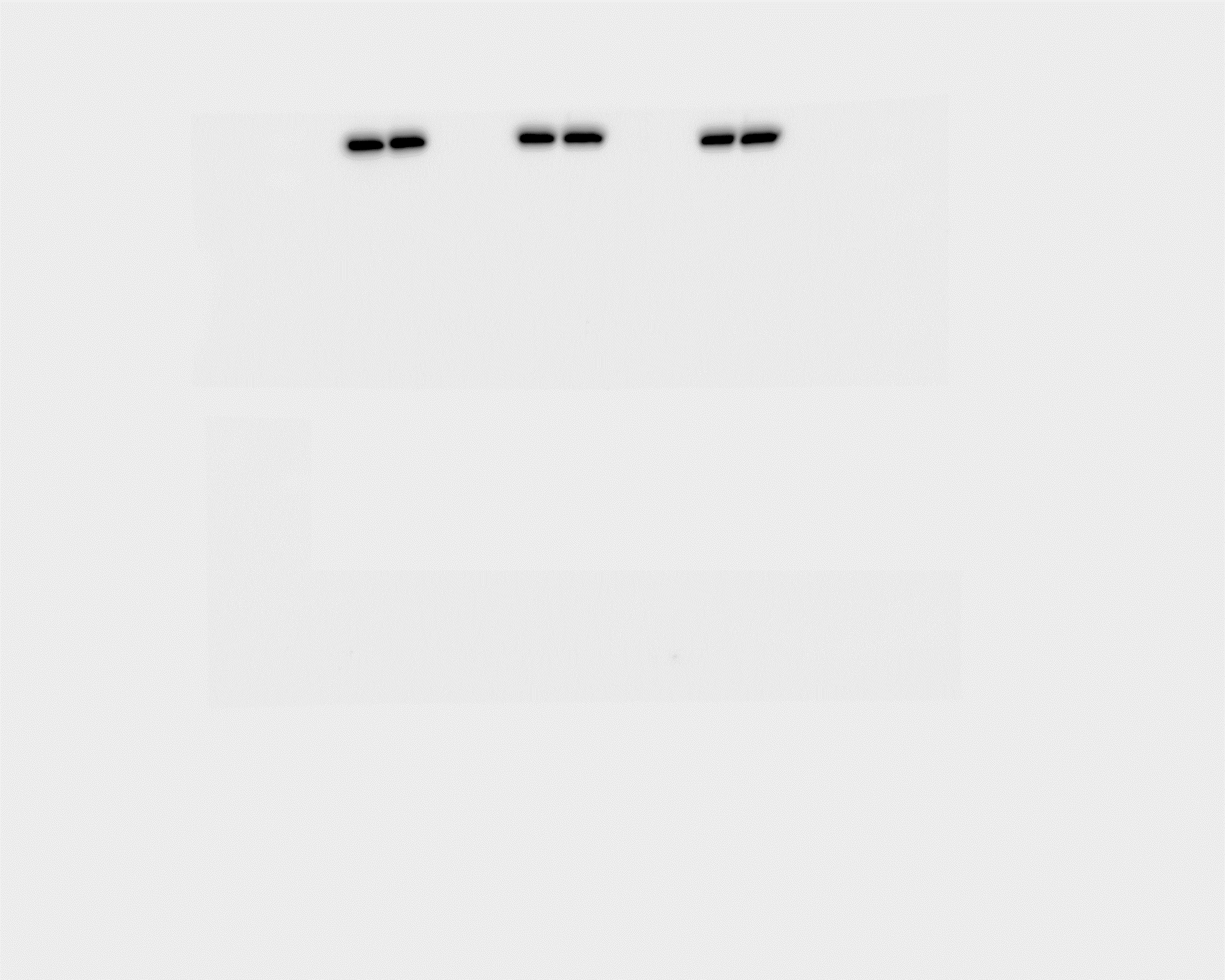

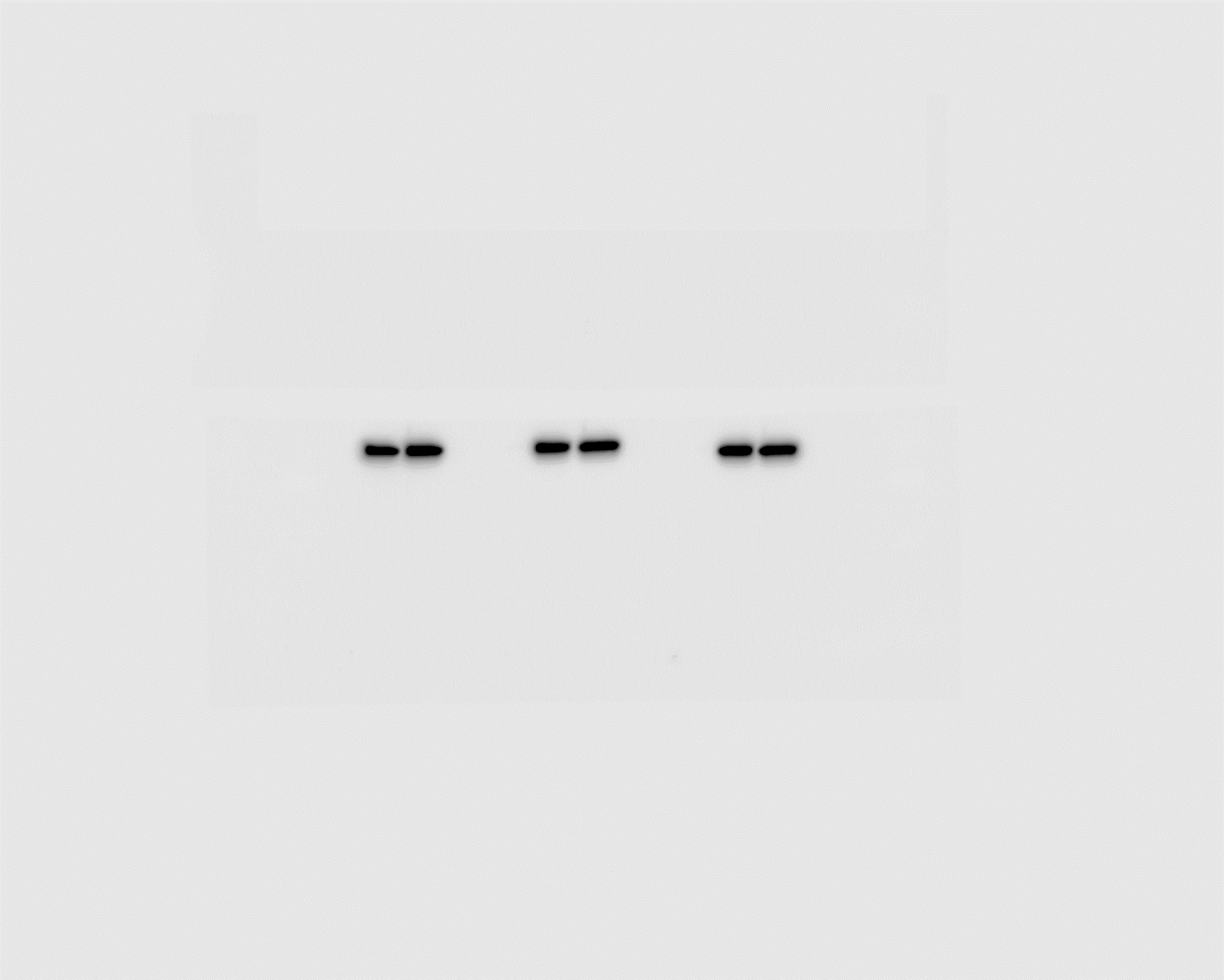

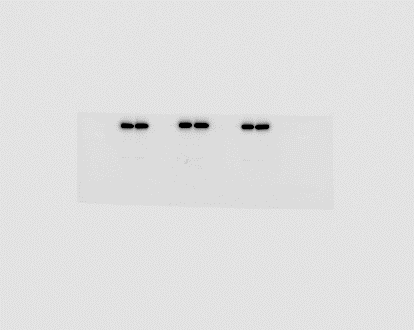

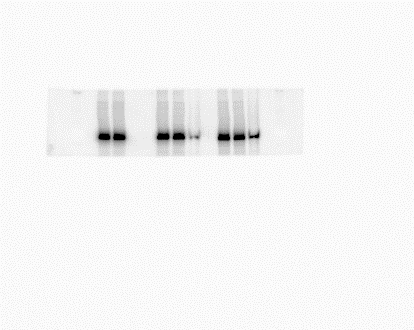

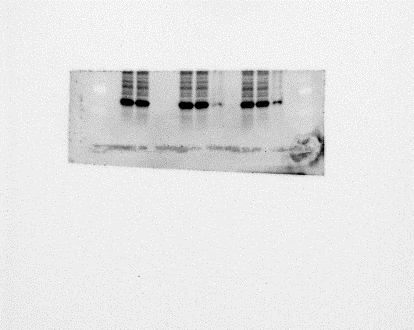

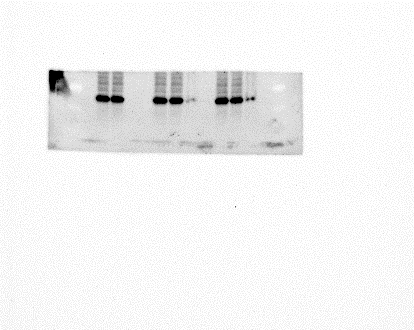

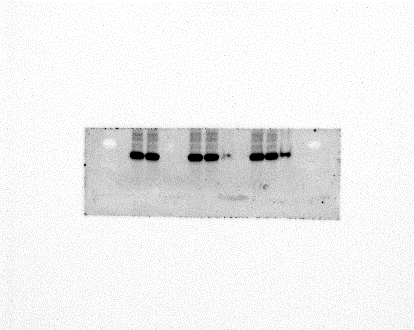

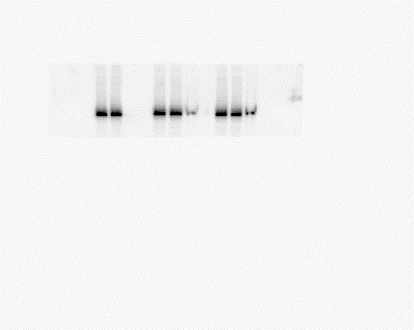

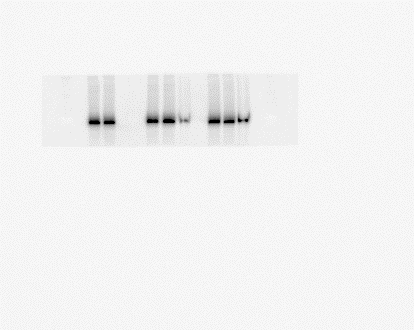


**Supplementary Fig. 2. Original blots for the binding of APT20TTMG to the U1 snRNP complex.** Human neuroblastoma SK-N-SH cells were lysed and prepared for protein immunoprecipitation, with quantifications normalized in relation to input (100%), and qRT-PCR immunoprecipitation assays, performed with RNA isolated from input (50%), flow-through - FT (50%), and eluate (100%) samples. Original Western blot images of GAPDH (**a**), U1-A (**b**), U1-70K (**c**), and U1-C (**d**). The first, second, and third replicates are represented in the lines one, two, and three, respectively.


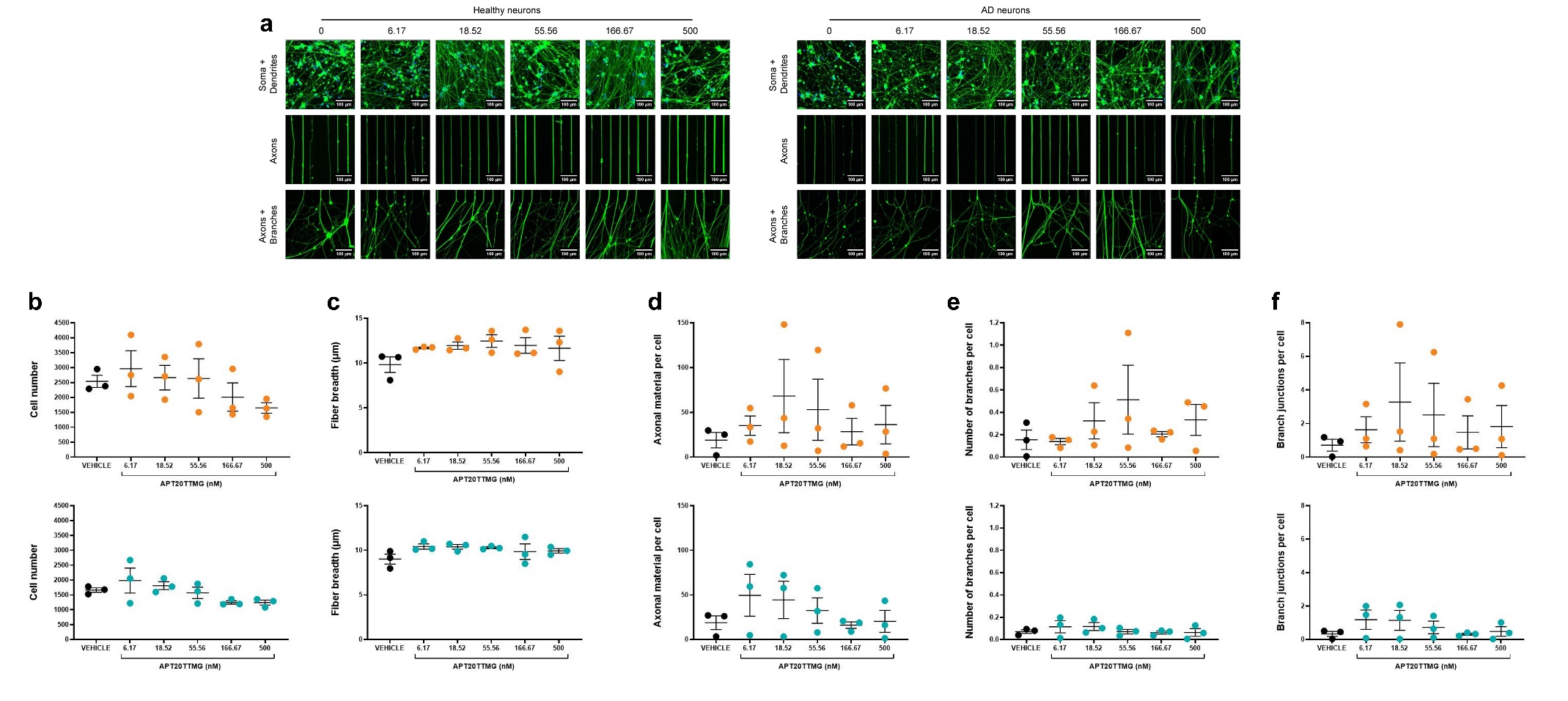


**Supplementary Fig. 3. APT20TTMG does not alter the morphological parameters of iPSC-derived neurons.** iPSC-derived healthy and AD neurons cultured for 6 days in NeuroHTSTM microplates were treated with five concentrations of APT20TTMG (6.17, 18.52, 55.56, 166.67, or 500 nM), for 7 days. Morphological parameters were evaluated in three different regions of the microplates at the end of treatment. Representative images of iPSC-derived healthy (left) and AD (right) neurons in the compartments of NeuroHTSTM microplates (**a**): upper (soma + dendrites), middle (axons), and bottom (axons + branches). Cell number (**b**), evaluated in the upper compartment. Fiber breadth (**c**), evaluated in the middle compartment. Axonal material per cell (**d**), number of branches per cell (**e**), and branch junction per cell (**f**), evaluated in the bottom compartments. Data are represented as mean ± SEM. Graphs show data from three independent experiments performed in up to three technical replicates, analyzed using ANOVA, followed by Tukey’s post hoc test, or the Kruskal-Wallis test, followed by Dunn’s post hoc test.


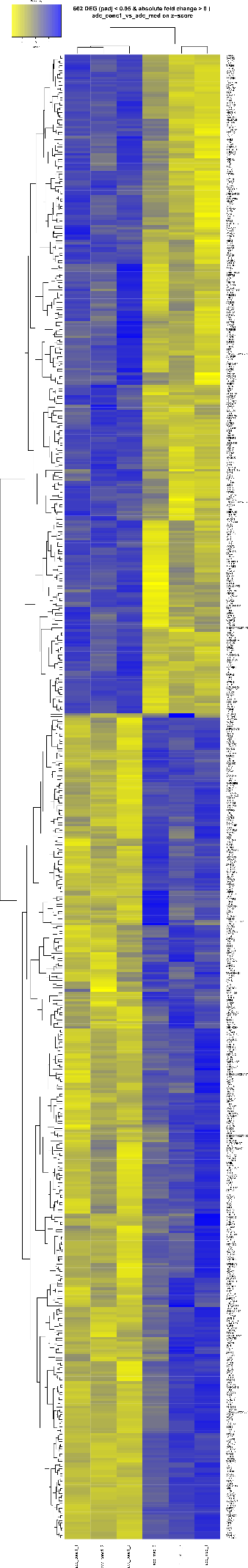

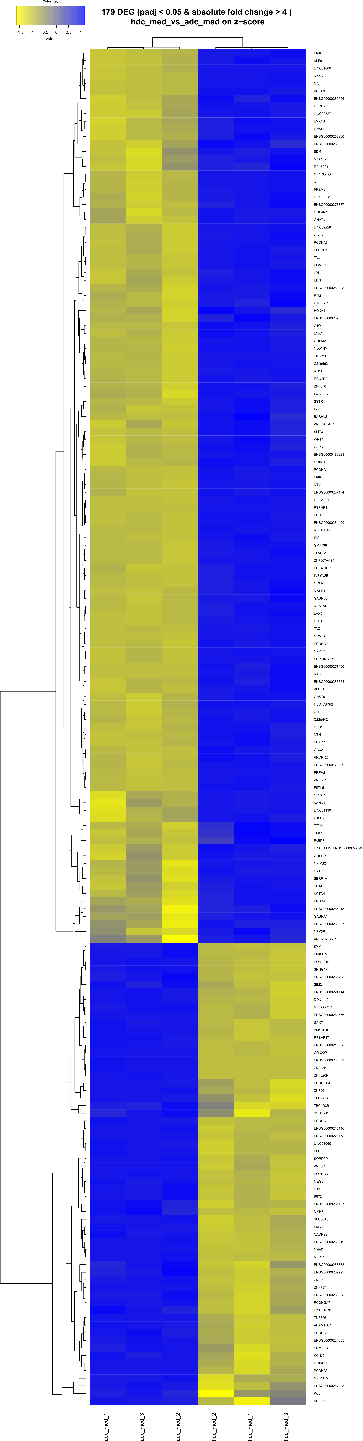


**a**

**b**

**Supplementary Fig. 4. Heat maps of differentially expressed genes in iPSC-derived neurons from healthy and AD patients.** (**a**) A total of 179 differentially expressed genes of non-treated healthy neurons in relation to non-treated AD neurons (medium only), considering as cut-off a Padj < 0.05 and Log fold change > 4. (**b**) A total of 602 differentially expressed genes from AD neurons treated with 18.52nM of APT20TTMG in relation to non-treated AD neurons (medium only), considering as cut-off a Padj < 0.05.


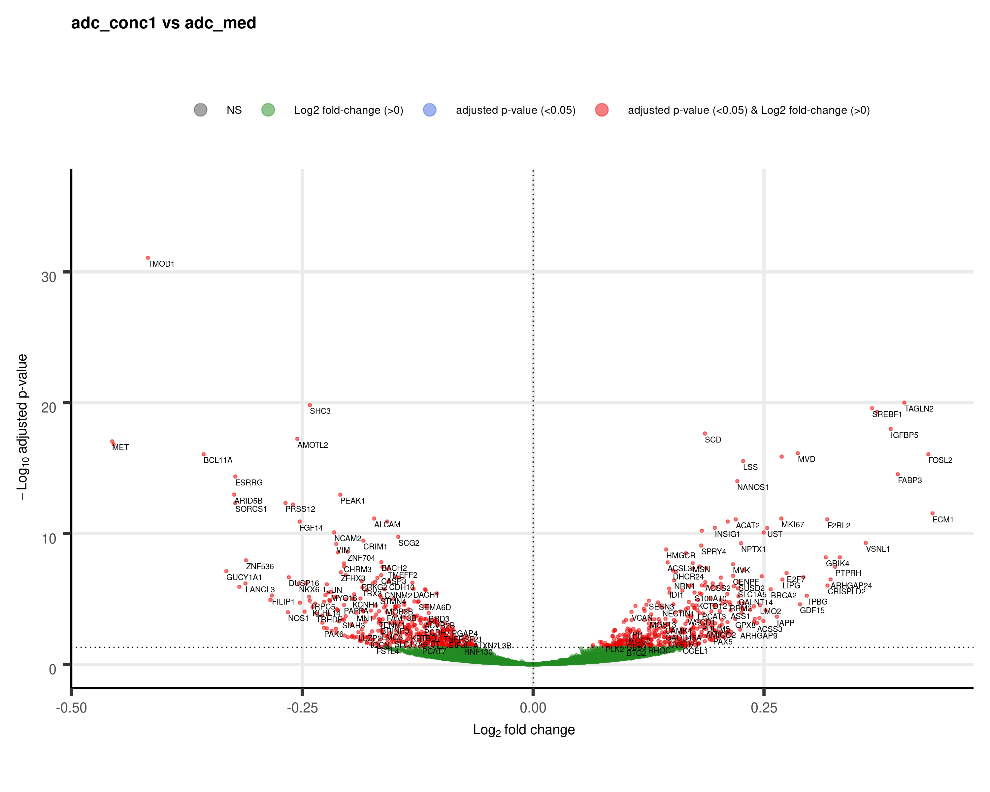

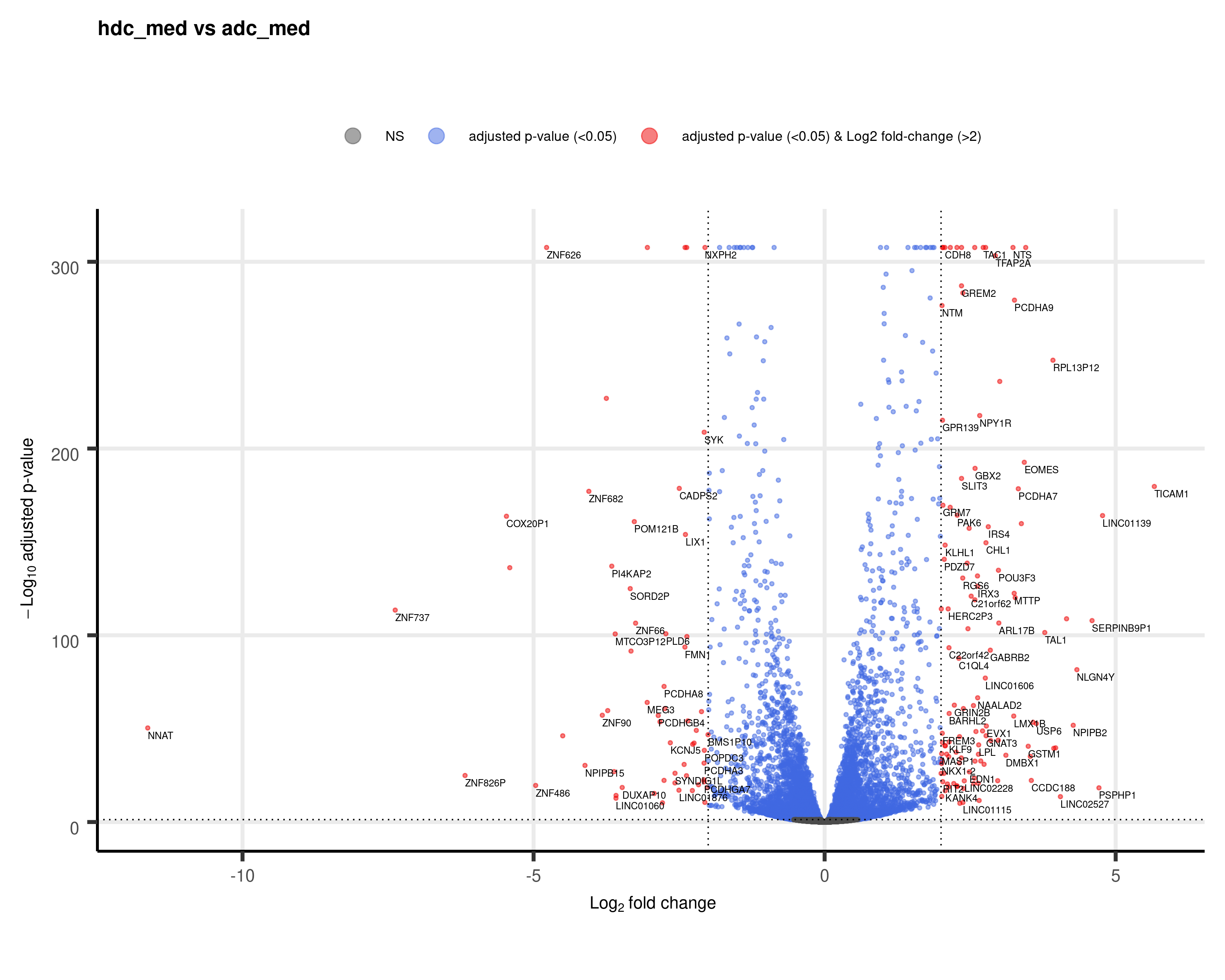


**a**

**b**

**Supplementary Fig. 5. Volcano plot of differentially expressed genes in iPSC-derived neurons from healthy and AD patients.** (**a**) Differentially expressed genes of non-treated healthy neurons in relation to non-treated AD neurons (medium only). (**b**) Differentially expressed genes from AD neurons treated with 18.52 nM of APT20TTMG in relation to non-treated AD neurons (medium only).


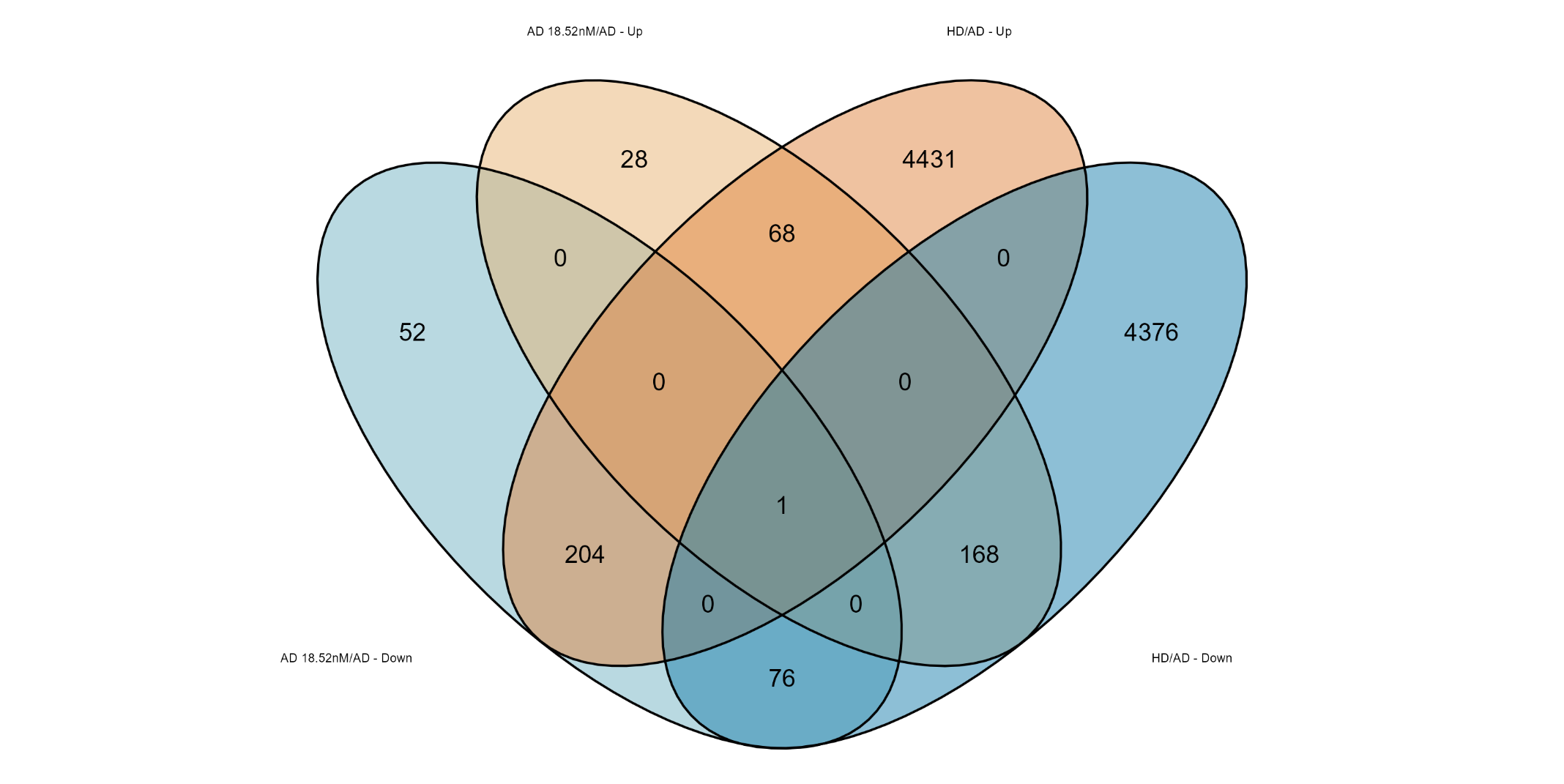


**Supplementary Fig. 6. Venn diagrams of all differentially expressed genes in iPSC-derived neurons from healthy and AD patients treated and non-treated with 18.52nM of APT20TTMG.** The Venn diagram shows the up and downregulated differentially expressed genes of AD neurons treated with 18.52 nM of APT20TTMG in relation to non-treated AD neurons (AD 18.52 nM/AD) versus non-treated healthy neurons in relation to non-treated AD neurons (HD/AD).


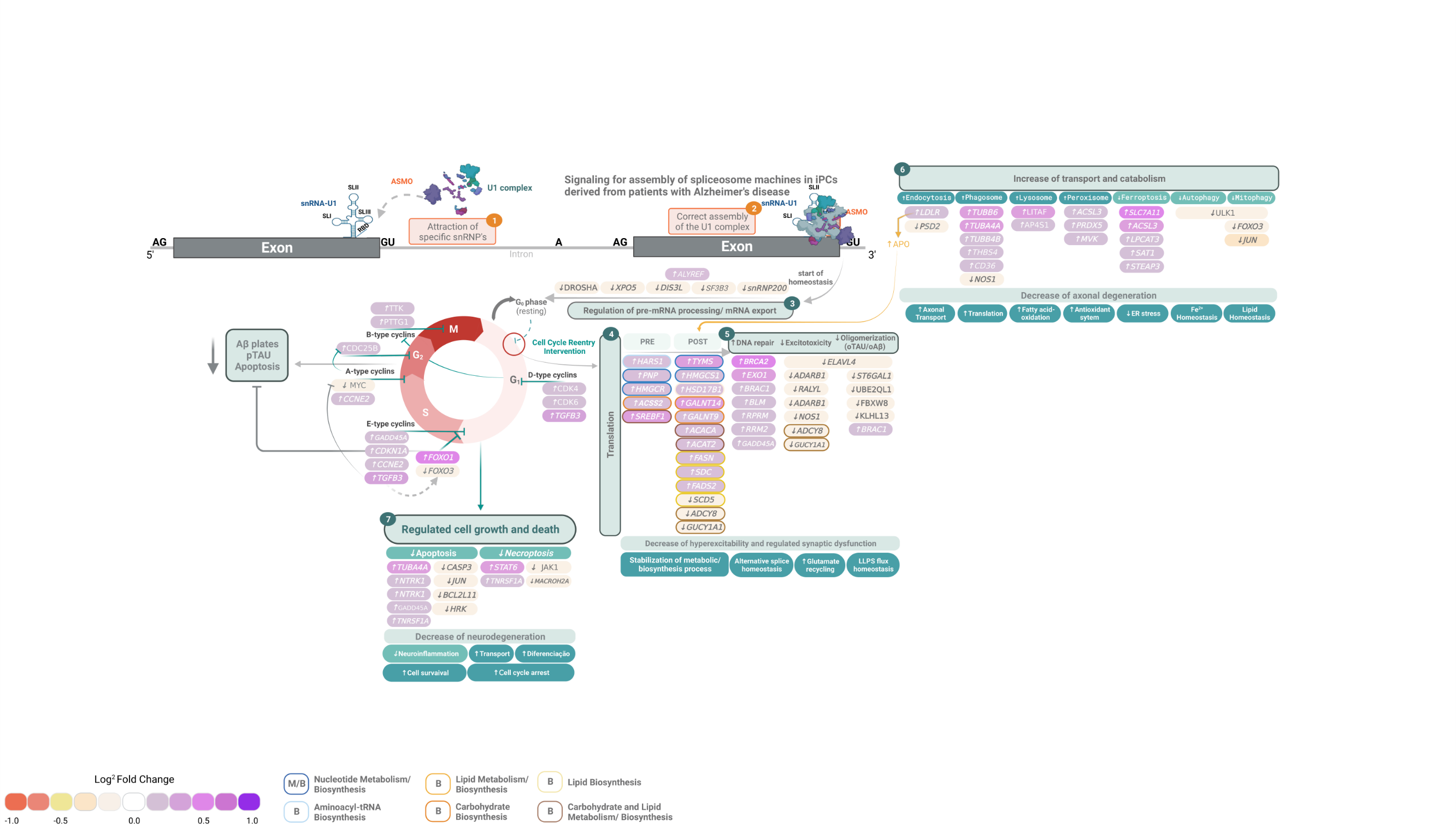

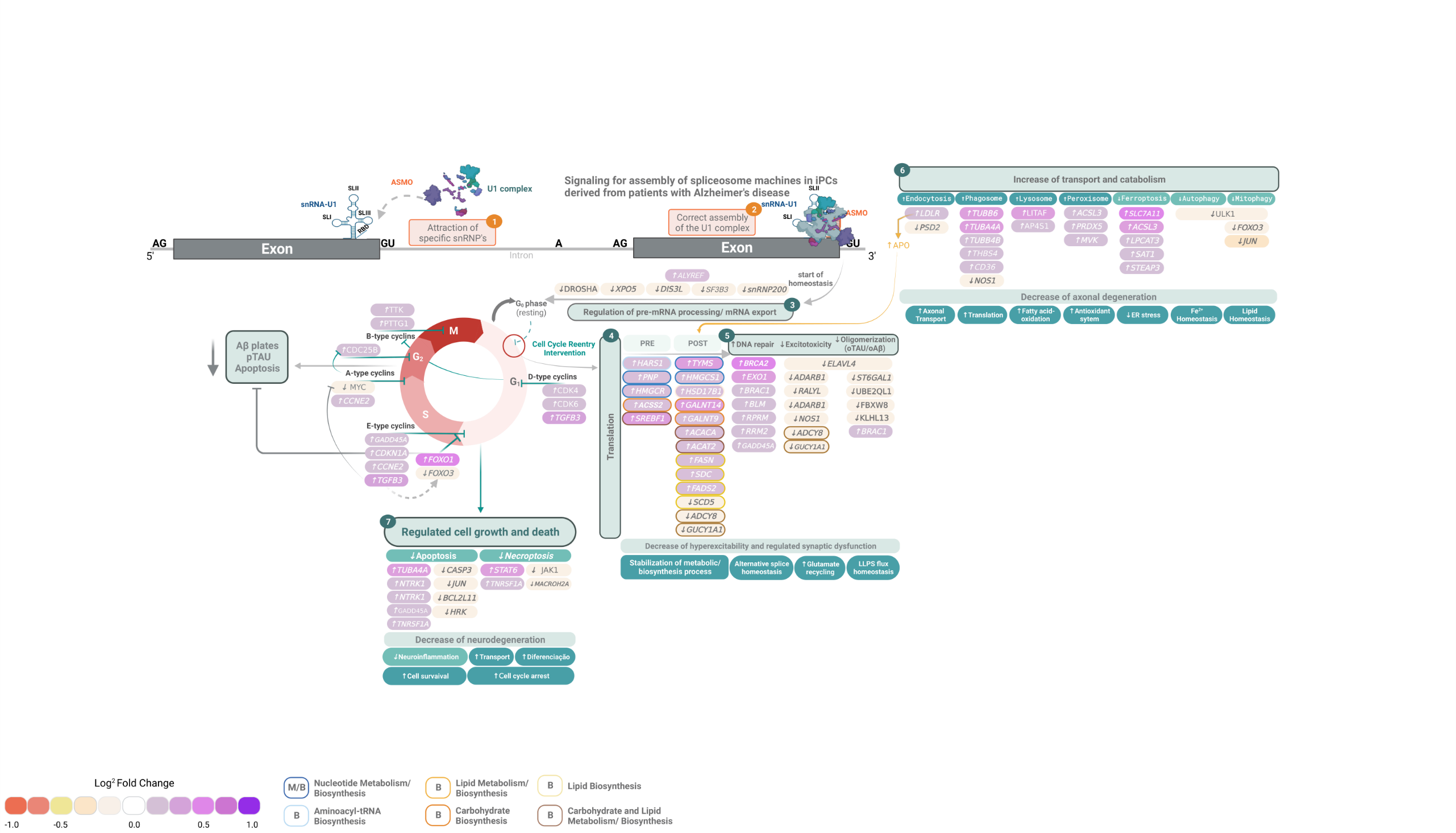


**Supplementary Fig. 7. Proposed mechanism of action of APT20TTMG on the U1 snRNP complex in iPSC-derived neurons.** The binding of APT20TTMG to U1 snRNP complex (1) attracts snRNPs and (2) corrects U1 assembly, leading to splicing homeostasis. As a result, it (3) regulates pre-mRNAs and mRNAs export, (4) modulates the expression of genes related to pre-and post-translational events, (5) upregulates genes related to DNA repair, (6) increases protein transport and catabolism, and (7) decreases axonal degeneration and regulates cell growth and death. DEGs are represented by rounded rectangles with fill colors representing the log2FoldChange: -1.0 (red), -0.5 (yellow), 0.0 (white), 0.5 (pink), and 1.0 (purple). Roles of DEGs are represented by outlines of these rounded rectangles with different colors: Nucleotide metabolism/biosynthesis (dark blue), aminoacyl-tRNA biosynthesis (light blue), lipid metabolism/biosynthesis (light orange), carbohydrate biosynthesis (dark orange), lipid biosynthesis (light purple), and carbohydrate and lipid metabolism/biosynthesis (dark purple). Created with BioRender.com.


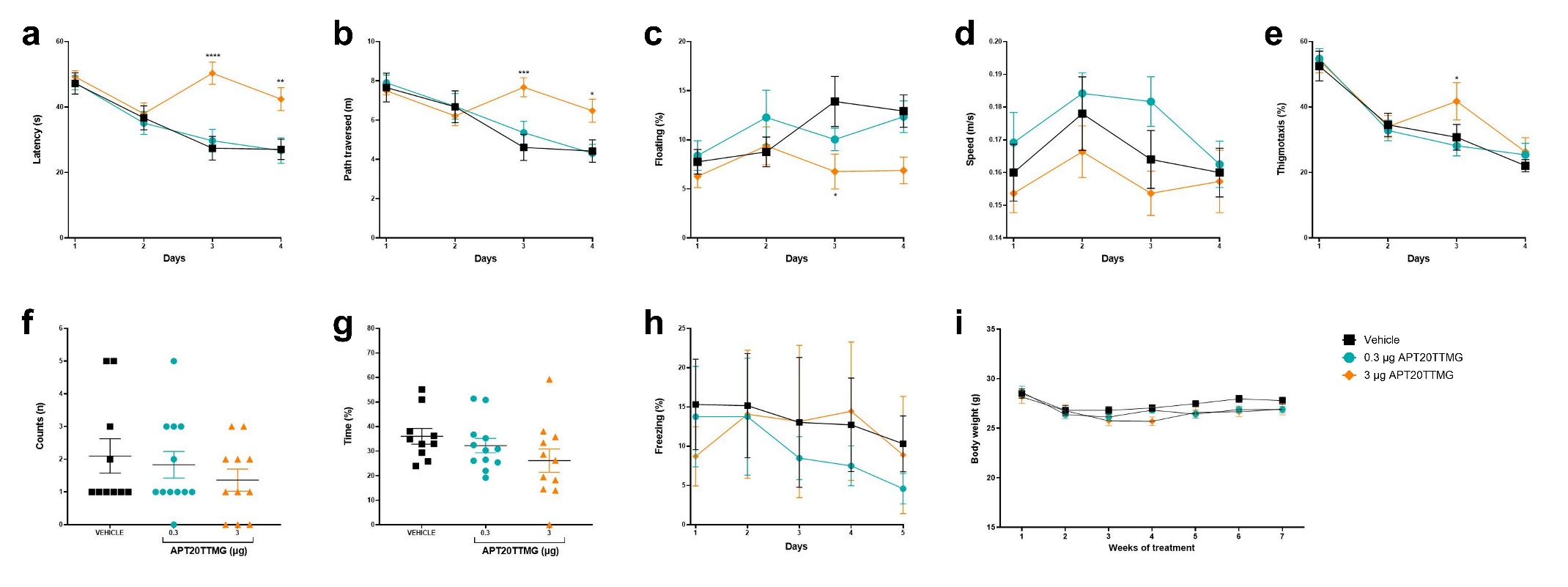


**Supplementary Fig. 8. A low dose of APT20TTMG does not induce cognitive changes in SAMP8 mice.** Female SAMP8 mice were evaluated in the Morris water maze (MWM) and contextual fear conditioning (CFC) tests in the fifth and sixth weeks, respectively, of the 42-day treatment with APT20TTMG (0.3 µg/day or 3 µg/day) or vehicle. Escape latency (**a**), distance traversed (**b**), floating (**c**), velocity (**d**), and thigmotaxis behavior (**e**), recorded daily for four consecutive training days in the MWM test. Number of target zone crossings (**f**) and abidance in the target quadrant (**g**), analyzed during the probe trial on day 5 of the MWM test. Freezing behavior over time in the CFC test (**h**). Body weight, evaluated throughout 7 weeks (**i**). Data are represented as mean ± SEM (n = 10–12). Graphs were analyzed using two-way ANOVA, followed by Tukey’s multiple comparisons test (a-e, h, i), Kruskal-Wallis test, followed by Dunn’s test (f), and one-way ANOVA, followed by Dunnett's test (g).


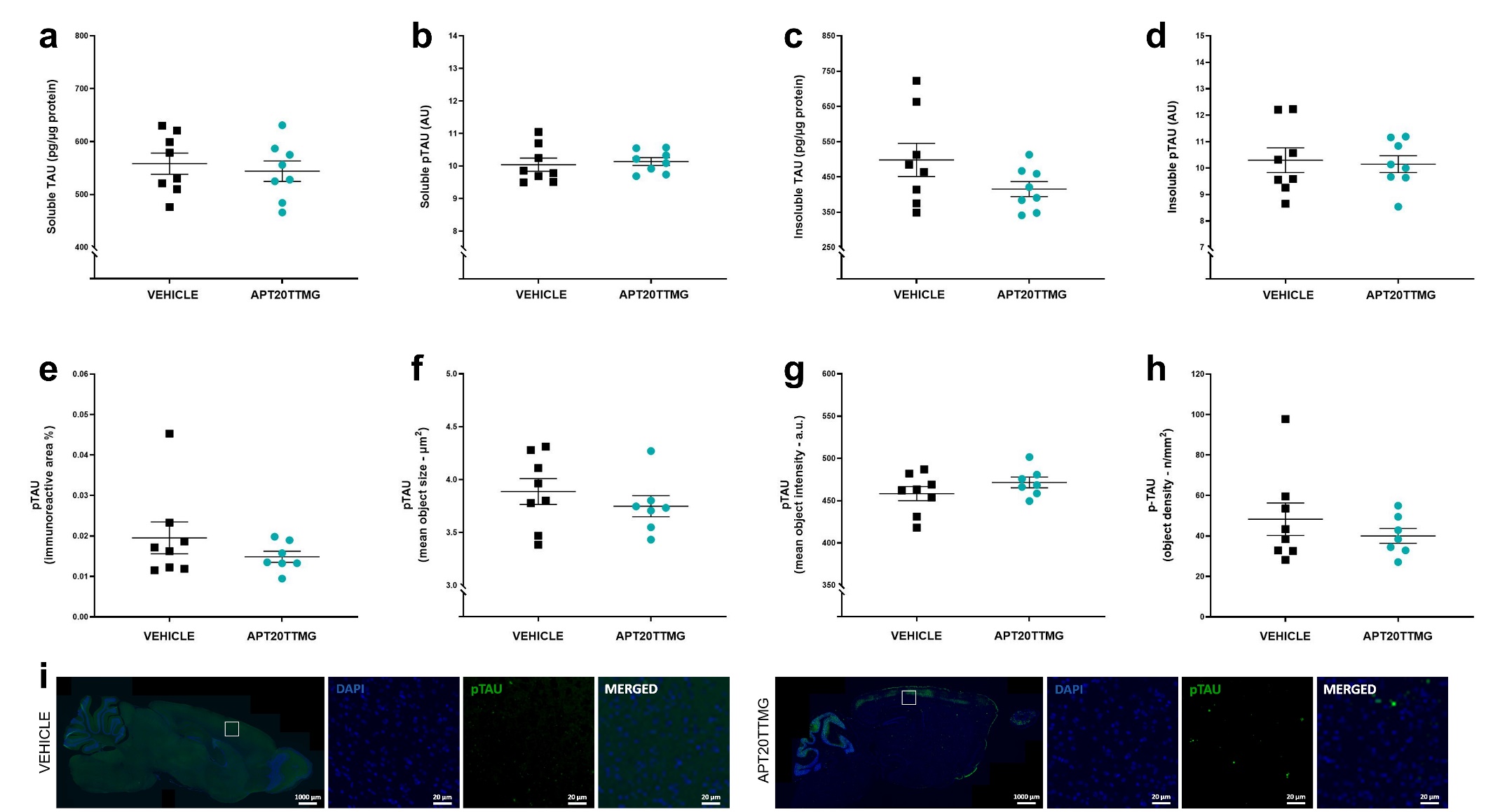


**Supplementary Fig. 9. APT20TTMG does not alter levels of soluble and insoluble TAU and pTAU (T231) or pSer202/pThr205-TAU immunofluorescence in the cerebral cortex of SAMP8 mice.** Following a 42-day treatment with 0.3 µg/day APT20TTMG or vehicle, female SAMP8 mice brains were collected and the cortex processed for ELISA and immunohistochemistry. Levels of soluble TAU (**a**), soluble pTAU (**b**), insoluble TAU (**c**), and insoluble pTAU (**d**) in the cortex. Immunoreactive area (**e**), mean object size (**f**), mean object intensity (**g**), mean object density (**h**) in the cortex, and representative image of cortex, double-labeled with pTAU (cytoplasm) and DAPI (nucleus) (**i**). The means of immunofluorescent signal were measured within the regions of interest (ROI) on 5 brain sections per mouse. Data are represented as mean ± SEM (n = 8 for ELISA and 7–8 for immunohistochemistry). Graphs were analyzed using t-test, comparing all groups to vehicle-treated control animals.


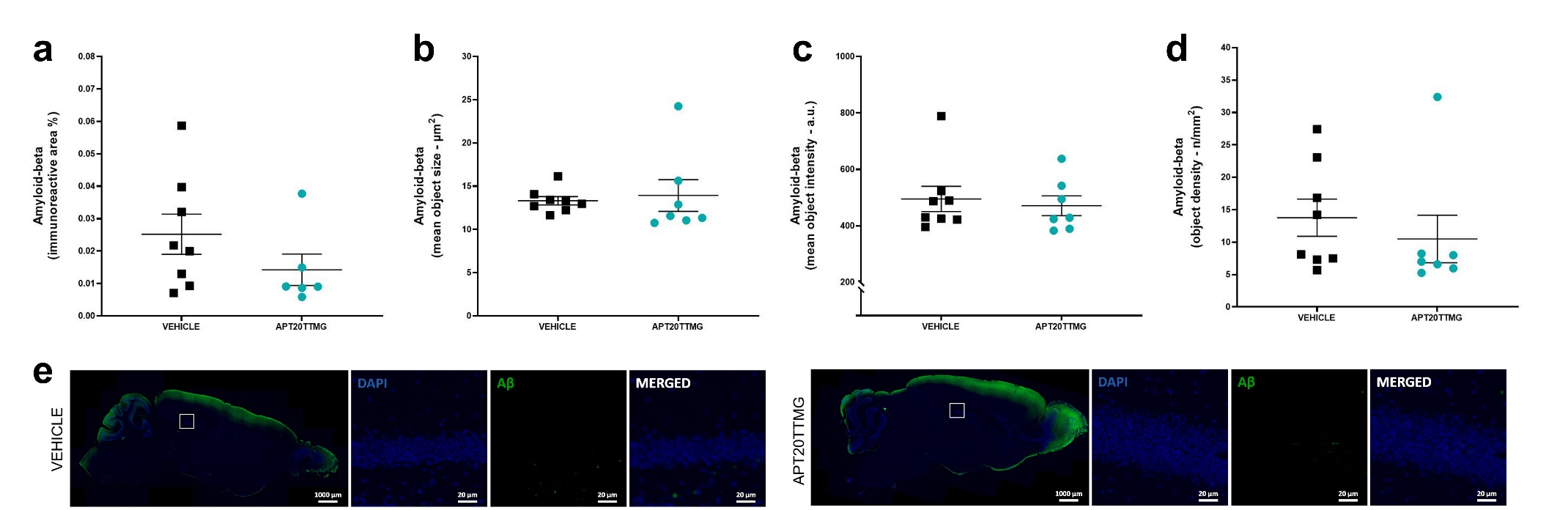


**Supplementary Fig. 10. APT20TTMG does not alter amyloid-beta (Aβ) immunofluorescence in the hippocampus of SAMP8 mice.** Following a 42-day treatment with 0.3 µg/day APT20TTMG or vehicle, female SAMP8 mice brains were collected and the hippocampus was processed for immunohistochemistry. Immunoreactive area (**a**), mean object size (**b**), mean object intensity (**c**), mean object density (**d**) in the hippocampus, and representative image of hippocampus, double-labeled with Aβ (cytoplasm) and DAPI (nucleus) (**e**). The means of immunofluorescent signal were measured within the regions of interest (ROI) on 5 brain sections per mouse and data are represented as mean ± SEM (n = 6–8). Graphs were analyzed using Mann-Whitney test (a and d) or T-test (b and c), comparing all groups to vehicle-treated control animals.


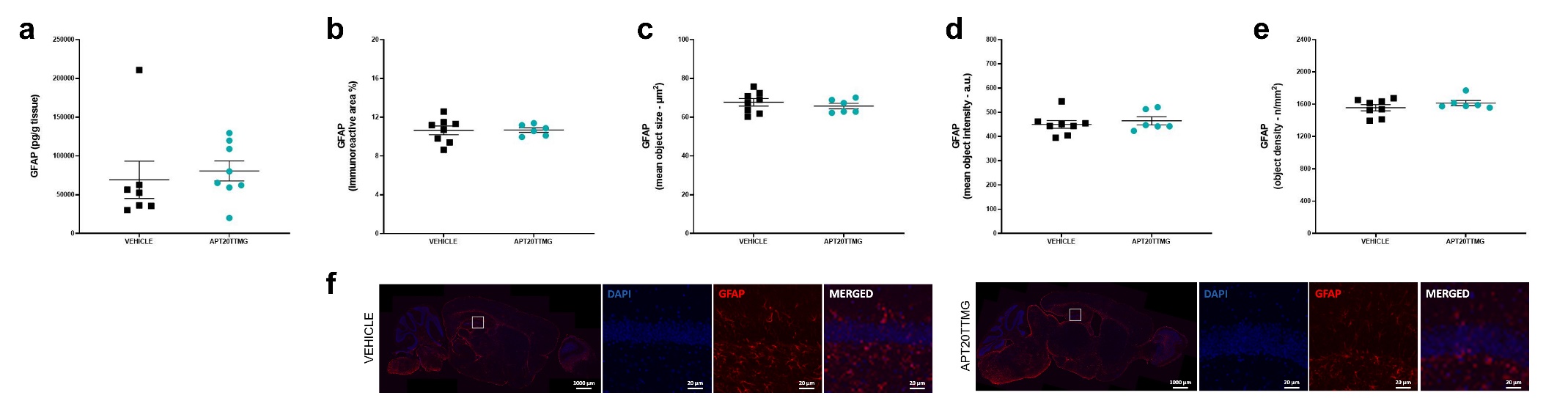


**Supplementary Fig. 11. APT20TTMG does not alter levels of GFAP in the hippocampus of SAMP8 mice**. Following a 42-day treatment with 0.3 µg/day APT20TTMG or vehicle, female SAMP8 mice brains were collected and the hippocampus processed for ELISA, in the soluble fraction, and for immunohistochemistry. GFAP levels, measured by ELISA (**a**). Immunoreactive area (**b**), mean object size (**c**), mean object intensity (**d**), mean object density (**e**) of GFAP immunofluorescence in the hippocampus, and representative image of hippocampus, double-labeled with GFAP (cytoplasm) and DAPI (nucleus) (**f**). The means of immunofluorescent signal were measured within the regions of interest (ROI) on 5 brain sections per mouse. Data are represented as mean ± SEM (n = 6–8 for ELISA and 6–8 for immunohistochemistry). Graphs were analyzed using Mann-Whitney (a) or T-test (b-e), comparing all groups to vehicle-treated control animals.


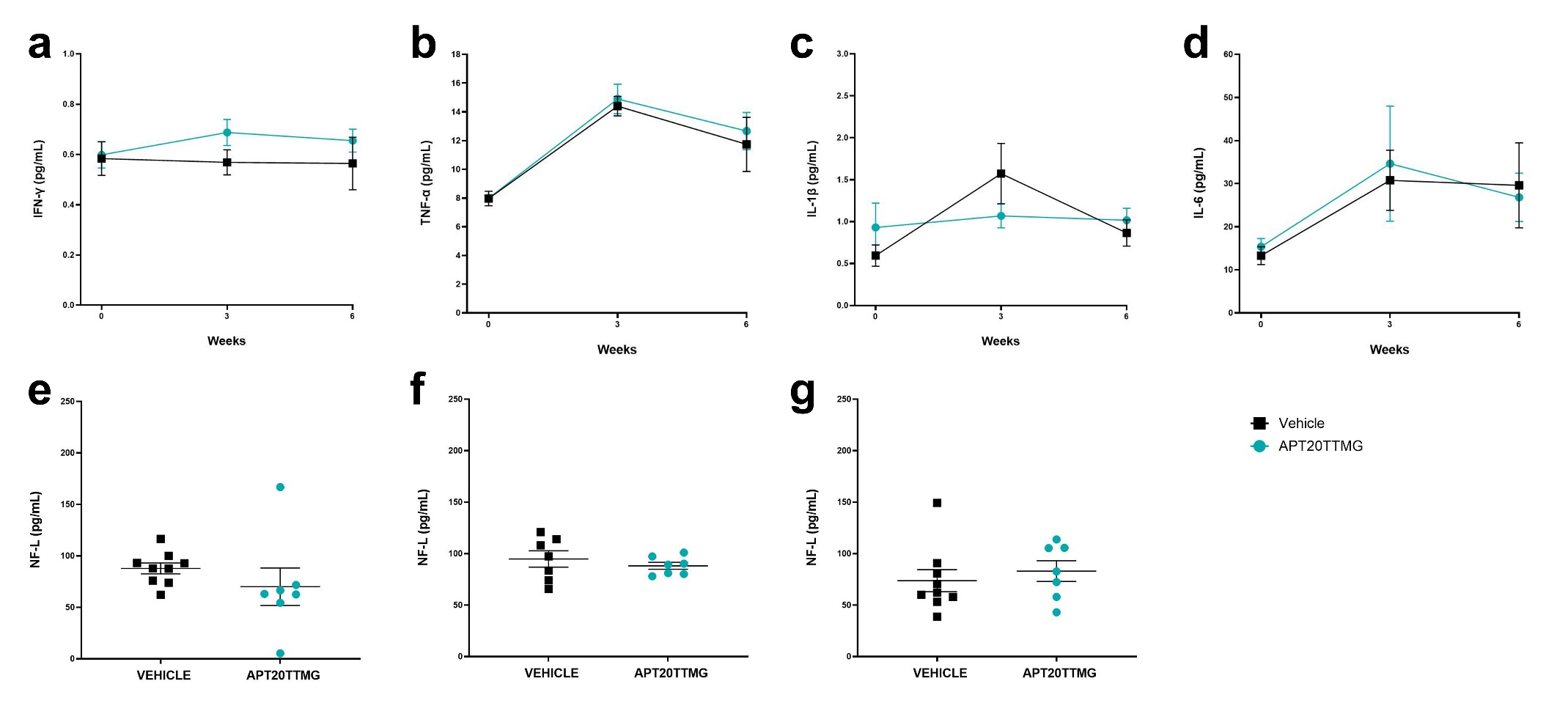


**Supplementary Fig. 12.  Plasmatic proinflammatory cytokines or neurofilament light chain (NF-L) levels are not altered by APT20TTMG in SAMP8 mice.** Proinflammatory cytokines and NF-L levels were measured by ELISA in *in vivo* (0 and 3 weeks) and in terminal blood plasma (6 weeks) of female SAMP8 mice treated with 0.3 µg/day APT20TTMG or vehicle for 42 days. Levels of IFN-γ (**a**), TNF-α (**b**), IL-1β (**c**), and IL-6 (**d**) in *in vivo* and terminal blood plasma. Levels of NF-L at baseline (prior to treatment start) (**e**), after 3 weeks of treatment (**f**), and after 6 weeks of treatment (terminal blood plasma) (**g**). Data are represented as mean ± SEM (n = 7–9). Graphs were analyzed using two-way ANOVA, followed by Tukey's multiple comparisons test (a-d), and Mann-Whitney test (e) or t-test (f and g), comparing all groups to vehicle-treated control animals.


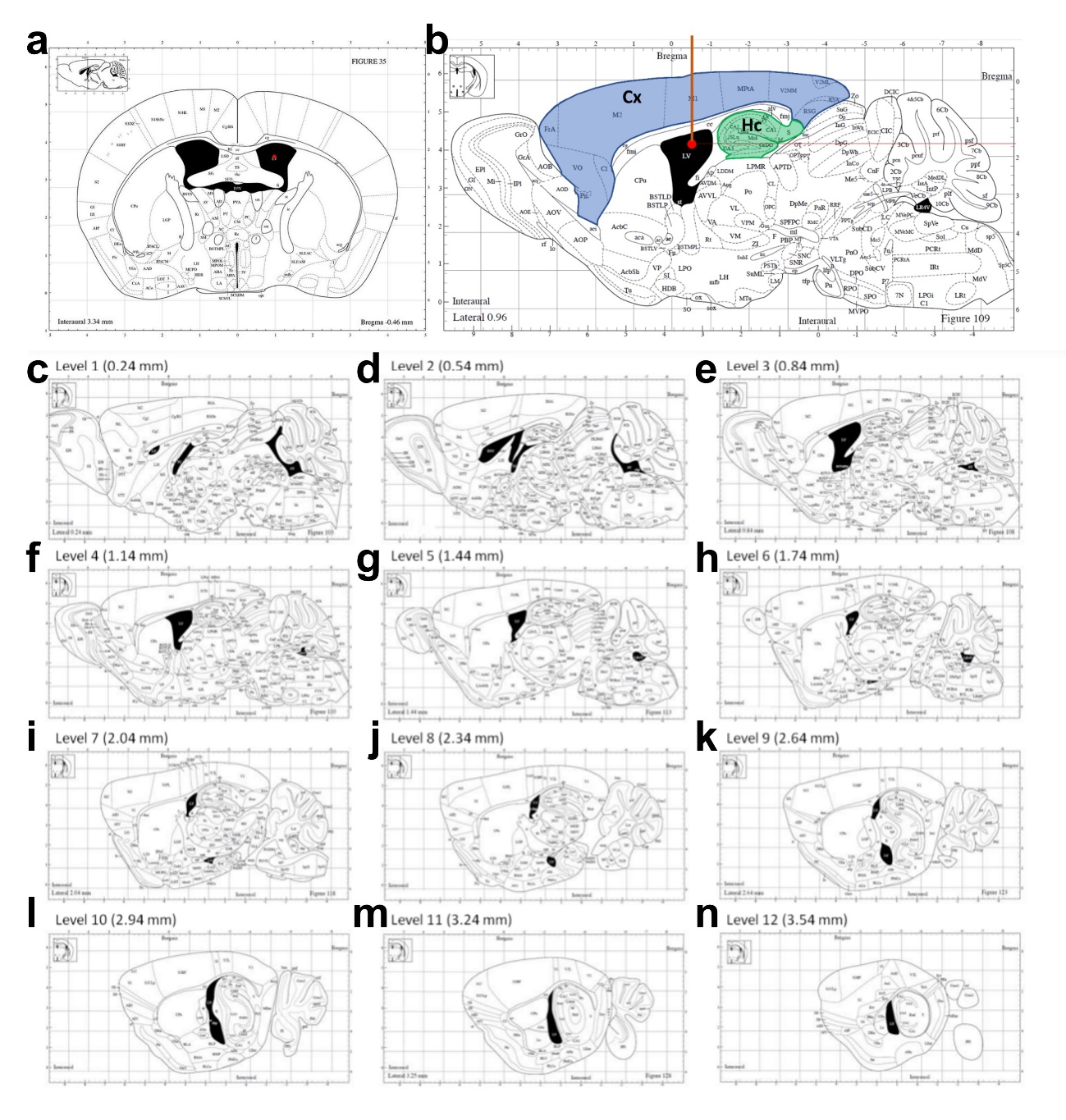


**Supplementary Fig. 13.  Coordinates for brain infusion cannula placement and histological brain sectioning levels in female SAMP8 mice.** Injection coordinates (anterior/posterior =  -0.5 mm, midline = 1.0 mm, and dorsoventral = 1.7 mm, from dura mater) are indicated by a red dot on both a coronal section (**a**) and a sagittal view (**b**). Approximate medio-sagittal levels obtained following the sectioning protocol (**c-n**). Drawings were taken from Paxinos & Franklin (2001), showing the stereotaxic coordinates.


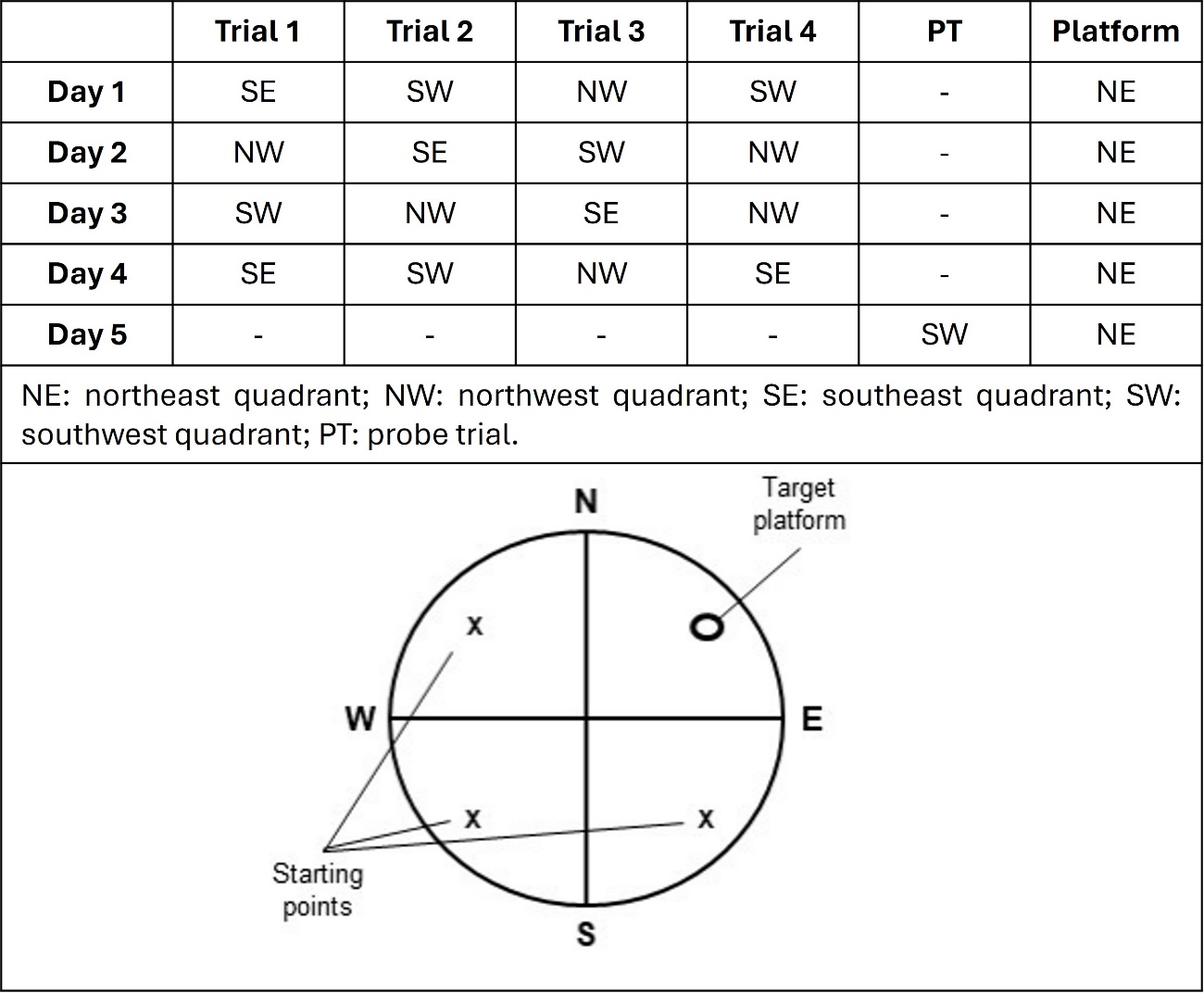


**Supplementary Fig. 14.** **Start positions for the Morris water maze (MWM) test.** Female SAMP8 mice were evaluated in the MWM test in the fifth week of the 42-day treatment with APT20TTMG (0.3 µg/day or 3 µg/day) or vehicle. During four consecutive days, animals were released from different start positions and the platform was located in the Northeast quadrant.
